# Social attention to activities in children and adults with autism spectrum disorder: effects of context and age

**DOI:** 10.1186/s13229-020-00388-5

**Published:** 2020-10-19

**Authors:** Dzmitry A. Kaliukhovich, Nikolay V. Manyakov, Abigail Bangerter, Seth Ness, Andrew Skalkin, Matthew S. Goodwin, Geraldine Dawson, Robert L. Hendren, Bennett Leventhal, Caitlin M. Hudac, Jessica Bradshaw, Frederick Shic, Gahan Pandina

**Affiliations:** 1 Janssen Pharmaceutica NV, Turnhoutseweg 30, 2340 Beerse, Belgium; 2 Janssen Research & Development, LLC, 1125 Trenton-Harbourton Road, Titusville, NJ 08560 USA; 3 Datagrok, INC, 1800 JFK Blvd Suite 300 PMB 90078, Philadelphia, PA 19103 USA; 4 312E Robinson Hall, Department of Health Sciences, Bouvé College of Health Sciences, Northeastern University, 360 Huntington Avenue, Boston, MA 02115 USA; 5 Duke Center for Autism and Brain Development and Duke Institute for Brain Sciences, Duke University School of Medicine, 2608 Erwin Road, Suite 30, Durham, NC 27705 USA; 6 Benioff Children’s Hospital, University of California, San Francisco, 401 Parnassus Avenue, Langley Porter, San Francisco, CA 94143-0984 USA; 7 Center for Youth Development and Intervention, University of Alabama, Box 870348, Tuscaloosa, AL 35487-0348 USA; 8 Department of Psychology, University of South Carolina, 1512 Pendleton Street, Columbia, SC 29201 USA; 9 Department of Pediatrics, Seattle Children’s Research Institute, Center for Child Health, Behavior and Development, University of Washington, 6200 NE 74th Street, Ste 110, Seattle, WA 98115-8160 USA

**Keywords:** Autism spectrum disorder, Social attention, Activity monitoring, Eye tracking, Biomarkers

## Abstract

**Background:**

Diminished visual monitoring of faces and activities of others is an early feature of autism spectrum disorder (ASD). It is uncertain whether deficits in activity monitoring, identified using a homogeneous set of stimuli, persist throughout the lifespan in ASD, and thus, whether they could serve as a biological indicator (“biomarker”) of ASD. We investigated differences in visual attention during activity monitoring in children and adult participants with autism compared to a control group of participants without autism.

**Methods:**

Eye movements of participants with autism (*n* = 122; mean age [SD] = 14.5 [8.0] years) and typically developing (TD) controls (*n* = 40, age = 16.4 [13.3] years) were recorded while they viewed a series of videos depicting two female actors conversing while interacting with their hands over a shared task. Actors either continuously focused their gaze on each other’s face (mutual gaze) or on the shared activity area (shared focus). Mean percentage looking time was computed for the activity area, actors’ heads, and their bodies.

**Results:**

Compared to TD participants, participants with ASD looked longer at the activity area (mean % looking time: 58.5% vs. 53.8%, *p* < 0.005) but less at the heads (15.2% vs. 23.7%, *p* < 0.0001). Additionally, within-group differences in looking time were observed between the mutual gaze and shared focus conditions in both participants without ASD (activity: *Δ* = − 6.4%, *p* < 0.004; heads: *Δ* = + 3.5%, *p* < 0.02) and participants with ASD (bodies: *Δ* = + 1.6%, *p* < 0.002).

**Limitations:**

The TD participants were not as well characterized as the participants with ASD. Inclusion criteria regarding the cognitive ability [intelligence quotient (IQ) > 60] limited the ability to include individuals with substantial intellectual disability.

**Conclusions:**

Differences in attention to faces could constitute a feature discriminative between individuals with and without ASD across the lifespan, whereas between-group differences in looking at activities may shift with development. These findings may have applications in the search for underlying biological indicators specific to ASD.

*Trial registration* ClinicalTrials.gov identifier NCT02668991.

## Background

There is a need for quantifiable and objective measures of behavior in autism spectrum disorder (ASD) that can aid diagnosis and stratification and may be useful as biomarkers (i.e., biological indicators of condition) for treatment response [1]. Given the phenotypic heterogeneity in ASD, eye tracking might be an objective way to assess social attention [2] wherein social attention differences have been noted in infant siblings of children with ASD, as well as toddlers, children, and adults with ASD, compared to individuals without ASD [3–8]. Current evidence suggests it may be possible to predict social-communicative outcomes and risk for ASD based on early visual attention to social stimuli, highlighting social attention as a potential predictive biomarker for ASD [9–16].

In addition to their use as predictive or diagnostic biomarkers, biosensors, such as eye trackers, have the potential to serve as a potential biomarker of change in ASD for example in clinical trials. Given the variability in when individuals are diagnosed and enrolled in interventions [17–19], it is critical to generate biomarkers at early (i.e., ≤ 3 years) and later developmental stages in order to generate developmentally appropriate biomarkers that can capture the changes and success of interventions across the lifespan. For this purpose, eye-tracking paradigms need to be tested for suitability and validity in the populations for which they are intended to be used. For greatest applicability, this means examining feasibility and utility across a range of clinically validated populations and across a wide array of age groups. It is well-known that social attention is abnormal across development for individuals with ASD [20, 21], replication of findings using consistent methodology across the heterogeneity of ASD remains limited [17, 21, 22], which may hamper our ability to establish a biomarker of social attention. Moreover, the extent to which observed differences are driven by use of differing experimental paradigms and methodologies across studies is also unclear. Finally, eye tracking may also be biologically relevant, as visual attention of this type may be related to social neural networks and thus not simply a behavioral phenomenon [23].

### Activity monitoring paradigm

One important role of social attention relates to selective attention toward the joint activities of others (i.e., activity monitoring). Toddlers with ASD have been found to monitor the activity of others less than their TD peers when observing a child and adult play interaction in short dynamic scenes [24]. Reduced attention to activity can lead to decreased opportunities for observational learning, which can impact social and cognitive development and have deleterious long-term effects [25, 26]. It also appears that complexity of features in the environment may compete with social stimuli, increasing the likelihood that abnormal patterns of attention are observed in individuals with ASD [21]. For instance, in a recent study of activity monitoring in ASD, toddlers were shown a set of stimuli that differed across three dimensions of interest: gaze direction of the actors, presence of background distractors, and dynamic nature of the stimuli [27, 28]. Consistent with prior work, findings show that toddlers with ASD, when compared to control toddlers, attended less to scenes overall, looked less at the activity and faces, and looked more at the background. Differences in activity monitoring between toddlers with ASD and other groups were most striking when background distractors were included and when stimuli were shown as dynamic videos. In contrast, gaze direction of the actors did not significantly influence between-group differences. Interestingly, unlike toddlers with ASD, older 3-year-old children with ASD did not show limited activity monitoring; however, like the toddlers, they showed decreased looking at heads and increased looking at background. An unanswered question is whether there are continuing developmental transitions by which children with ASD diverge from those without ASD in their viewing patterns toward activity scenes, which would impact the use of eye tracking as a biomarker across the lifespan.

### Current study

In the current study, we used an activity monitoring paradigm to investigate allocation of visual attention to stimuli in complex social scenes involving shared activities in older children and adults with ASD, and a typically developing (TD) comparison group. There is no current published literature describing differences between older children and adults with and without ASD on this task, and as such this work provides an upward extension of results in toddlers and at the preschool-age [27, 28]. We focused on the experimental manipulation of gaze direction (i.e., shared gaze focus on the activity versus mutual gaze between actors) and used only dynamic stimuli with background distractors.

Our primary aim was to establish preliminary data to aid in determining the utility of this paradigm as a discriminative biomarker in older individuals with and without ASD. Hypotheses based on previous findings [24] were that there would be less attention to the heads of actors, less attention to activity, and more attention to background distractors in the ASD group compared to the TD group. We also hypothesized, that response to gaze direction would be different between the ASD and TD groups, which in turn would modulate attention patterns both in these older children with ASD [29–31] and TD groups. Finally, in an exploratory analysis, we investigated whether differences in allocated attention during activity monitoring were related to individual differences in age, autism severity, and intelligence quotient (IQ).

## Methods

### Ethical practices

The Institutional Review Board at each of the nine participating study sites approved the study protocol and subsequent amendments. The study was conducted in accordance with the ethical principles of the Declaration of Helsinki, consistent with Good Clinical Practices and applicable regulatory requirements. Participants, their parents (for participants < 18 years old), or legally authorized representatives provided written informed consent before joining the study. Assent was obtained from any participants aged < 18 who were capable of understanding the nature of the study, and this was written assent for those who were able to write.

### Participants

Participants in the ASD group were aged ≥ 6 years with a confirmed diagnosis of ASD based on clinical examination, caregiver interview, and use of the Autism Diagnostic Observation Schedule, 2nd Edition (ADOS-2) [32]. Key exclusion criteria were a measured composite score on the Kaufmann Brief Intelligence Test-2 (KBIT-2) [33] of < 60, and history of or current significant medical illness. TD controls were aged ≥ 6 years with a score in the normal range on the Social Communication Questionnaire [34] who did not meet criteria for any major mental health disorder [35] as assessed using the Mini-International Neuropsychiatric Interview [36]. Age 6 was used as the cutoff for this study, since it is the lower regulatory age bound for clinical studies in psychiatry. Note that the KBIT-2 was only collected for individuals with ASD but not for TD controls. Participants were enrolled within the framework of a large, observational, multi-center study that was conducted from July 6, 2015, to October 14, 2016, at nine study sites in the USA (trial registration no. NCT02668991 at https://clinicaltrials.gov) and consisted of multiple passive viewing tests [37–42].

In total, 136 individuals with ASD and 41 TD controls completed the study. Out of those, after exclusions due to technical or calibration failures, 122 individuals with ASD and 40 TD controls were included for the activity monitoring test (Table 1, Additional file 8: Table S8).

**Table 1:** Participant characteristics

Characteristic	ASD (*n* = 122)	TD (*n* = 40)
Sex, *n* (%)		
Male	93 (76.2)	26 (65.0)
Female	29 (23.8)	14 (35.0)
*χ*^2^ test, *p* value	0.16	
Age		
Mean (SD)	14.5 (8.0)	16.4 (13.3)
Median (range)	12 (6–54)	11.5 (6–63)
Kolmogorov–Smirnov test, *p* value	0.73	
ADOS-2 total score, mean (SD, range)	7.6 (1.7, 4–10)	–
KBIT-2 IQ composite score, mean (SD, range)	98.6 (19.9, 60–136)	–

*n* indicates the number of participants

*ASD* autism spectrum disorder, *ADOS-2* Autism Diagnostic Observation Schedule, 2nd Edition, *KBIT-2* Kaufmann Brief Intelligence Test-2, *TD* typically developing

### Activity monitoring task

**Fig. 1:**
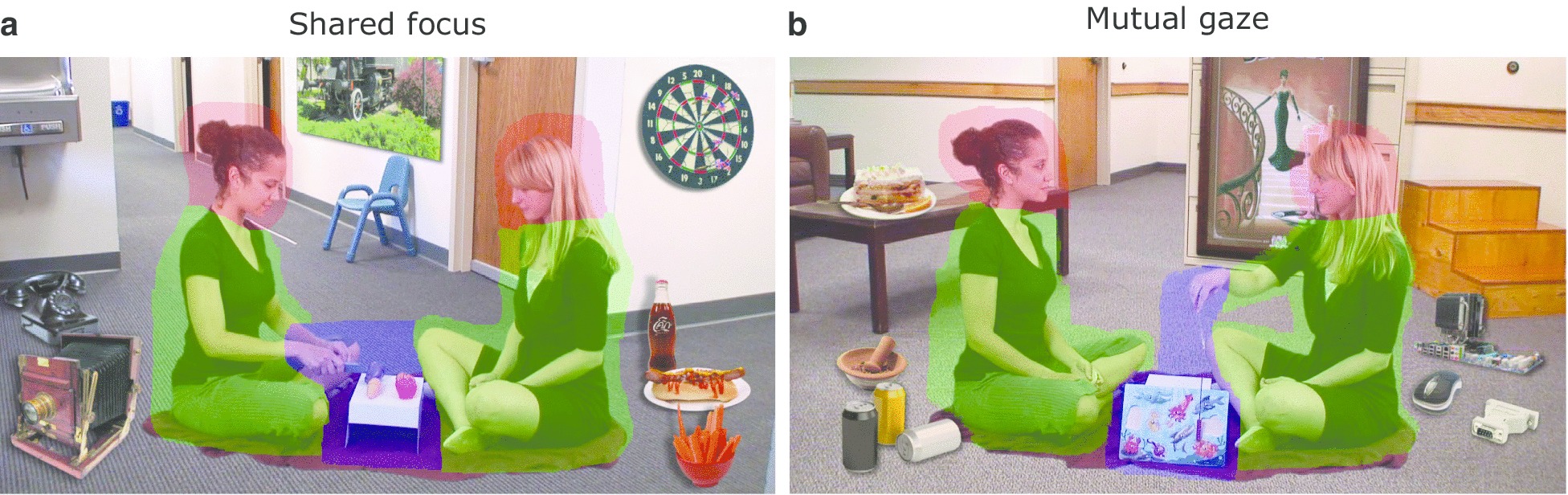
Stimulus conditions. A *Shared focus* condition. B *Mutual gaze* condition. The regions uniformly filled in different colors correspond to different ROIs: red, *Heads*; green, *Bodies*; blue, *Activity*. The remaining part of a visual scene corresponds to the ROI *Background*. Note that the three color masks were invisible during actual experiments and are presented here for the sake of illustration only. *ROI* region-of-interest

Participants viewed a series of videos presenting two female actors involved in a shared activity. In each video, the actors were viewed in profile with bodies facing each other and hands interacting over a shared task. The actors were placed in a typical office environment with barren walls and carpet that was enriched by visually salient distractors, including furniture, food, electronic and mechanical devices. Throughout videos, the actors were exclusively and continuously focusing either on each other’s face (*Mutual gaze* condition) or the shared activity area (*Shared focus* condition) while performing a simple action (e.g., cutting vegetables) and talking to each other (Fig. 1). The conversation between the actors involved simple language to accommodate participants with limited language.

Each participant viewed four videos in total (two each of *Shared focus* and *Mutual gaze* conditions). Each video lasted 20 s. The presentation order of the two stimulus conditions was random across participants.

### Procedure

Participants sat in a comfortable chair approximately 60 cm from a 23-inch computer screen (1920 × 1080 pixels). The height of the chair and screen were adjusted to ensure that participants’ eyes were level with the center of the screen. Eye-tracking data were collected using a 30 Hz Tobii X2 eye tracker mounted below the screen. iMotions Biometric Research Platform (https://imotions.com/) was used for stimuli presentation, data synchronization, and automatic calibration. Participants could freely observe presented stimuli. Before each experimental period, a five-point calibration procedure consisting of animated cartoon characters paired with an auditory cue was performed.

### Behavior rating scales

Parents or caregivers of individuals with ASD reported on the following domains: ASD symptoms (Autism Behavior Inventory [ABI] [43, 44]); problem behaviors (Aberrant Behavior Checklist—Community [ABC] [45, 46]); emotional and behavioral disorders (Child Adolescent Symptom Inventory [CASI] [47]); social impairment (Social Responsiveness Scale 2™ [SRS-2] [48]); and repetitive behaviors (Repetitive Behavior Scale—Revised [RBS-R] [49]). Each of the above scales and ADOS-2 consist of subscales that reflect different clusters of ASD symptoms (see Additional file 1: Table S1).

### Data analysis

Standard region-of-interest (ROI) analysis techniques were adapted for the analysis of gaze patterns (Fig. 1). The examined ROIs included the shared *Activity* area, the *Bodies*, and *Heads* of the two actors in a video, and the remaining *Background*. The videos were designed such that no major movements of ROIs occurred. Time spent by a participant looking at a specific ROI was normalized by the total viewing time for each video separately and averaged across videos per stimulus condition. The average percentage of time spent by a participant looking at stimuli relative to their presentation duration is referred to as the level of visual attention in that condition. Overall level of visual attention was obtained by averaging levels across the two stimulus conditions per participant. A two-sided, two-sample Kolmogorov–Smirnov test was used to compare the level of visual attention between the two groups of participants.

A linear mixed-effects model (widely implemented for multi-level data in clinical trials [50]) was used to compare % looking time between the two groups of participants and stimulus conditions for each individual ROI. Each model included the percentage of time spent looking at a specific ROI as a dependent variable, with stimulus condition, participant group, participant’s age and sex as fixed effects. Each model additionally included an interaction between stimulus condition and participant group. To account for within-participant variability in % looking time, each model included a participant identifier as a random intercept. The R package “nlme” was used to fit the models. Each model was fit by maximizing the restricted log-likelihood function. Significance of fixed effects was assessed using analysis of variance type III sum of squares and the Wald *χ*^2^ test (see Additional file 2: Table S2), as implemented in the R package “car.” Post hoc pair-wise comparisons were performed using the Tukey–Kramer correction for multiple comparisons. The least-squares mean estimates, their standard errors (SE), and two-sided 95% confidence intervals for different levels of the modeled categorical factors were obtained with the R package “lsmeans” (see Additional file 3: Table S3). This package was also used to run post hoc pair-wise comparisons (see Additional file 4: Table S4). The goodness of fit of each linear mixed-effects model was assessed by computing marginal and conditional coefficients of determination (*R*^2^) according to Nakagawa, Johnson, and Schielzeth (2017) and using the R package “MuMin” (see Additional file 5: Table S5 and Additional file 6: Table S6). For the sake of comparison with other studies, Cohen’s d was additionally computed for different combinations of stimulus condition and participant group for each individual ROI separately (see Additional file 4: Table S4). Alternatively, we also tested a linear mixed-effects model that was similar to that described above but included ROI and all its interactions with stimulus condition and participant group as additional fixed effects. The model was tested using the data of all ROIs, except for the ROI *Background*, to account for correlations between % looking time for different ROIs that existed due to normalization by the total viewing time (i.e., the sum of % looking time across the four ROIs was equal to 100% for each participant; see above). The same approach for post hoc pair-wise comparisons as described above was applied to this model. The outcomes of this model are reported in detail in Supplementary Material (see Additional file 12: Table S9, Additional file 13: Table S10, Additional file 14: Table S11, and Additional file 15: Figure S4).

A linear mixed-effects model was applied to compare slopes of the linear relationships between participant’s age and % looking time for the ROIs *Activity* and *Heads* between the two groups of participants. The model for each of these two ROIs included the percentage of time spent looking at that ROI as a dependent variable and participant’s age, group, and an interaction between age and group as fixed effects. A participant identifier served as the random intercept. The data for each ROI were pooled across the two stimulus conditions. The same approach as that described above was applied to test for statistical significance of the fixed effects (see Additional file 7: Table S7). Alternatively, to account for a potential effect of stimulus condition on the obtained results, we also tested a linear mixed-effects model that was similar to that described above but included stimulus condition and all its interactions with participant’s age and group as additional fixed effects. The model was tested separately for the ROI *Activity* and *Heads*. Moreover, to account for a potential effect of ROI, the latter model was further expanded to include ROI and all its interactions with stimulus condition, participant’s age and group as additional fixed effects. The model was tested using the data of both ROIs *Activity* and *Heads*. The outcomes of these models are reported in detail in Supplementary Material (see Additional file 16: Table S12 and Additional file 17: Table S13).

All reported correlations (*r*_S_) were Spearman partial correlations (given their lower susceptibility to potential outliers compared to Pearson correlations). Participant’s sex and age served as covariates for the computation of correlations between % looking time for different ROIs and the KBIT-2 IQ composite score in the group with ASD (see Additional file 9: Figure S1). The same list of covariates extended by the inclusion of KBIT-2 IQ composite score was used to compute correlations between % looking time for different ROIs and ASD symptoms severity (see Additional file 10: Figure S2 and Additional file 1: Table S1). Spearman partial correlation coefficients and corresponding two-sided *p* values were computed using the R package “ppcor.” No correction for multiple testing was performed for the computed correlation coefficients. Note that the number of statistical tests and, thus, the exact cutoff for significant *p* values in each analysis was debatable. For example, correction for multiple testing for the relationships between % looking time for different ROIs and ASD symptoms severity (Table 2) could have been done in multiple ways: for each behavior rating scale separately but across all ROIs and stimulus conditions, for each ROI separately but across all scales and stimulus conditions, or combining all tests regardless of behavior rating scale, ROI and stimulus condition. More options are available when accounting for individual ASD symptoms, as assessed by the behavior rating scales administered in the study (Additional file 1: Table S1). For the reasons outlined above, the *p* values were reported “as is,” with values < 0.05 considered significant.

**Table 2:** Correlations between % looking time for different ROIs and total score of behavior rating scales

Behavior rating scale	ROI		Activity	Background	Bodies	Heads
	Condition	*n*				
ABI	Shared focus	120	− 0.090 (0.33)	0.116 (0.21)	− 0.007 (0.94)	0.079 (0.40)
	Mutual gaze	107	* − 0.204 (0.04)*	0.031 (0.75)	0.042 (0.67)	0.099 (0.32)
ADOS-2	Shared focus	120	0.030 (0.75)	0.107 (0.25)	− 0.040 (0.67)	− 0.060 (0.52)
	Mutual gaze	107	0.097 (0.33)	0.029 (0.77)	0.015 (0.88)	* − 0.212 (0.03)*
CASI-Anx	Shared focus	120	− 0.083 (0.37)	0.051 (0.58)	− 0.017 (0.86)	0.151 (0.11)
	Mutual gaze	107	− 0.169 (0.09)	0.005 (0.96)	− 0.009 (0.93)	0.100 (0.31)
RBS-R	Shared focus	120	− 0.085 (0.36)	0.003 (0.98)	− 0.124 (0.18)	*0.227 (0.02)*
	Mutual gaze	107	− 0.169 (0.09)	0.003 (0.98)	− 0.057 (0.56)	0.137 (0.17)
SRS-2	Shared focus	119	− 0.059 (0.53)	0.077 (0.41)	− 0.021 (0.82)	0.053 (0.57)
	Mutual gaze	106	− 0.123 (0.22)	0.038 (0.70)	− 0.048 (0.63)	0.048 (0.63)

The data are presented for each of the two stimulus conditions separately. Cells contain Spearman partial correlation coefficients along with the corresponding two-sided *p* values in parentheses. The correlation coefficients are computed on the data of all participants with ASD, with participant’s age, sex, and KBIT-2 IQ composite score being used as covariates. Cells with *p* values below 0.05 are highlighted in italic. *n* indicates the number of participants

*ABI* Autism Behavior Inventory, *ASD* autism spectrum disorder, *ADOS-2* Autism Diagnostic Observation Schedule, 2nd Edition, *CASI-Anx* Child Adolescent Symptom Inventory—Anxiety, *KBIT-2* Kaufmann Brief Intelligence Test-2, *RBS-R* Repetitive Behavior Scale—Revised, *ROI* region-of-interest, *SRS-2* Social Responsiveness Scale 2

## Results

The two groups of participants did not significantly differ in their overall level of visual attention to presented stimuli (ASD vs. TD, mean [SE]: 87.2% [1.18%] vs. 91.5% [1.06%], *p* > 0.16). Similarly, level of visual attention did not vary between the groups in any of the two stimulus conditions when the latter were analyzed separately (both, *p* > 0.11) (see Additional file 11: Figure S3).

**Fig. 2:**
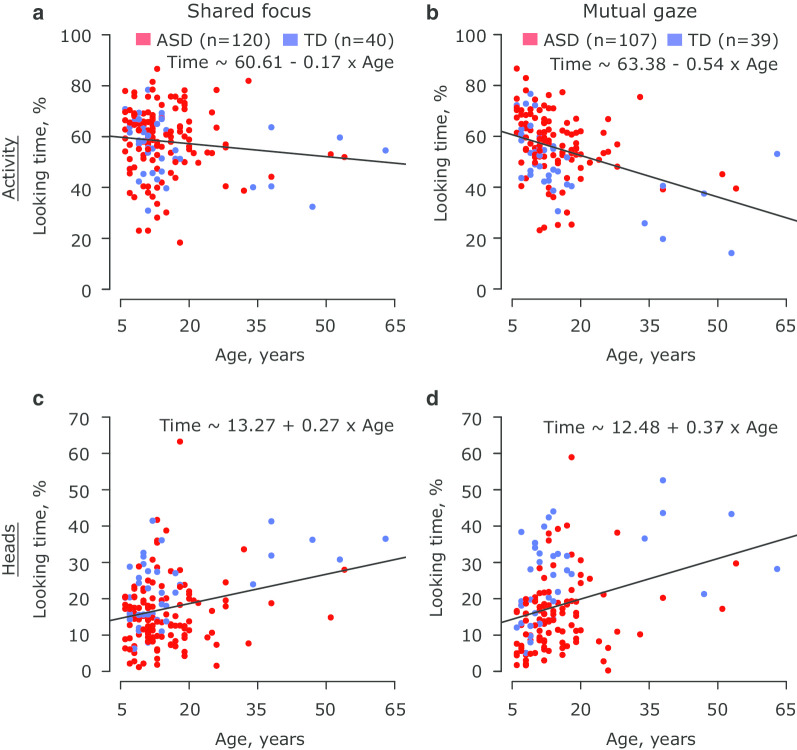
Scatter plots between participant’s age and looking time for the ROIs *Activity* and *Heads*. The data are presented for each stimulus condition (**a**, **c**: *Shared focus*; **b**, **d**: *Mutual gaze*) and ROI (**a**, **b**: *Activity*; **c**, **d**: *Heads*) separately. Dots denote individual participants. The red and blue colors correspond to the data of individuals with ASD and TD controls, respectively. n indicates the number of participants. The black line in each panel represents the best linear fit of the data pooled across the two groups of participants, with the equation for that fit being presented in the same panel. *ASD* autism spectrum disorder, *ROI* region-of-interest, *TD* typically developing

Figure 2 shows distributions of % looking time for each individual ROI, stimulus condition, and group of participants (see Additional file 20: Table S16 for statistics on individual sites). Linear mixed-effects models revealed a significant effect of participant group on looking time for the ROIs *Activity* (*p* < 0.005) and *Heads* (*p* < 0.0001) but no effect of stimulus condition (both, *p* > 0.06) (see Additional file 2: Table S2 and Additional file 4: Table S4). Specifically, individuals with ASD spent more time looking at *Activity* than TD controls (ASD vs. TD, mean [SE]: 58.5% [1.12%] vs. 53.8% [1.76%]); in contrast, individuals with ASD spent less time looking at *Heads* (15.2% [0.89%]) than TD controls (23.7% [1.39%]). In addition, both ROIs revealed a significant interaction between stimulus condition and participant group (*Activity*: *p* < 0.04; *Heads*: *p* < 0.005). The % looking time in individuals with ASD did not differ between the two stimulus conditions for both *Activity* (*Shared focus* vs. *Mutual gaze*, mean [SE]: 59.5% [1.23%] vs. 57.5% [1.27%], *p* > 0.25) and *Heads* (15.4% [0.94%] vs. 15.0% [0.96%], *p* > 0.92) (see Additional file 3: Table S3 and Additional file 4: Table S4). In contrast, stimulus condition significantly modulated % looking time in TD controls for each of these two ROIs. Specifically, TD controls spent significantly more time looking at *Activity* in the *Shared focus* than in the *Mutual gaze* stimulus condition (57.0% [1.97%] vs. 50.6% [1.98%], Cohen’s *d* = 0.51, *p* < 0.005), with the opposite observed for *Heads* (21.9% [1.50%] vs. 25.4% [1.51%], Cohen’s *d* = 0.33, *p* < 0.02). Lastly, the linear mixed-effects model showed a significant effect of stimulus condition on % looking time for the ROI *Bodies* (*p* < 0.0003). The latter effect was only observed in participants with ASD as a significant difference in % looking time between the two stimulus conditions (*Shared focus* vs. *Mutual gaze*, mean [SE]: 7.08% [0.55%] vs. 8.69% [0.56%], Cohen’s *d* = 0.23, *p* < 0.002). No significant fixed effects were observed for the ROI *Background*. All between-group comparisons remained statistically significant (all *p*’s < 0.05) in the alternative model that combined the data across ROIs and additionally included ROI and all its interactions with stimulus condition and participant group as fixed effects (see Additional file 12: Table S9, Additional file 13: Table S10, Additional file 14: Table S11, and Additional file 15: Figure S4).

**Fig. 3:**
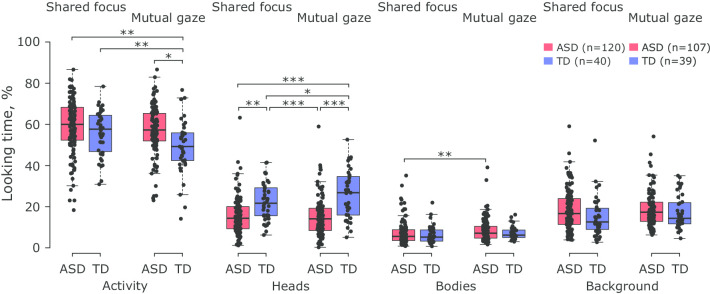
Distributions of % looking time for each individual ROI, stimulus condition, and group of participants. The data of each ROI, stimulus condition and group of participants (with autism—red, without autism—blue) are summarized in a form of boxplots. Black dots denote individual participants. n indicates the number of participants. *p value < 0.05, **p value < 0.005, ***p value < 0.0005 (corrected for multiple comparisons for each individual ROI using the Tukey–Kramer method; see Additional file 3: Table S3 and Additional file 4: Table S4). *ASD* autism spectrum disorder, *ROI* region-of-interest, *TD* typically developing

Participant’s age showed a significant effect on % looking time for the ROIs *Activity* (*p* < 0.0001) and *Heads* (*p* < 0.0001) (see Additional file 2: Table S2). Specifically, % looking time for *Activity* decreased with participant’s age, whereas the reverse was the case for *Heads* (Fig. 3). A different set of linear mixed-effects models was used (see Methods Section) to test for differences in slopes of the identified relationships between participant’s age and % looking time between the two groups of participants. As expected, the models again revealed a significant effect of participant’s age on % looking time for both ROIs *Activity* (*p* < 0.03) and *Heads* (*p* < 0.02) (see Additional file 6: Table S6 and Additional file 7: Table S7). However, no model showed a significant interaction between participant’s age and group (both *p*’s > 0.40), thus suggesting a similar strength of the identified relationships across the two groups of participants. Similarly, no alternative model that accounted for the effect of stimulus condition and ROI on the obtained results revealed a significant interaction between participant’s age and group (all *p*’s > 0.10; see Additional file 16: Table S12 and Additional file 17: Table S13), thus further confirming the findings reported above. To test whether a significant effect of participant’s age on % looking time for the ROIs *Activity* and *Heads* was driven by older participants (Fig. 3), the models described above were fitted using the data of all participants (1) below 40 years, (2) below 35 years, (3) below 30 years, (4) below 25 years, and (5) below 20 years separately (see Additional file 18: Table S14, Additional file 19: Table S15). No model revealed a significant effect of participant’s age on % looking time for the ROI *Activity* for any of the five tested data samples (all *p*’s > 0.09; Additional file 19: Table S15). Yet, participant’s age was significantly associated with % looking time for the ROI *Heads* for all data samples (all *p*’s < 0.05), except for that including the participants aged below 30 years (*p* = 0.0502). Remarkably, when analyzing the data of each stimulus condition separately, relationships between participant’s age and % looking time, as assessed by Spearman correlations, proved to be statistically significant for either ROI across the five tested age groups in the *Mutual gaze* (all *p*’s < 0.01; Additional file 18: Table S14) but not in the *Shared focus* (all *p*’s > 0.11) condition.

The data on participants’ intelligence, as assessed by KBIT-2, were collected only in participants with ASD. This precluded the use of these data in comparisons of % looking time between the two groups of participants. When relating % looking time for different ROIs to the KBIT-2 IQ composite score in participants with ASD, the KBIT-2 IQ score revealed significant negative correlations with % looking time for *Bodies* (*r*_S_ = − 0.189, *p* < 0.05) and *Heads* (*r*_S_ = − 0.208, *p* < 0.03) in the *Shared focus* stimulus condition (see Additional file 9: Figure S1). However, the same correlations failed to reach statistical significance (*Bodies*: *r*_S_ = − 0.097, *p* = 0.32; *Heads*: *r*_S_ = − 0.166, *p* = 0.09) in the *Mutual gaze* condition. No other significant correlations were observed (all *p* > 0.11).

To test whether overall severity of ASD symptoms manifested in percentage of time spent by individuals with ASD looking at a specific ROI, we correlated % looking time with the total score of behavior rating scales (*n* = 5). The correlation coefficients were computed for each ROI and stimulus condition separately, and the results of these computations are presented in Table 2. Of the 40 computed correlation coefficients, only three (7.5%) were significant. These included negative correlations in the *Mutual gaze* condition between the ABI core ASD symptom scale score and *Activity* (*r*_S_ = − 0.204, *p* < 0.04), the ADOS-2 total score and *Heads* (*r*_S_ = − 0.212, *p* < 0.03), as well as a positive correlation between the RBS-R total score and *Heads* (*r*_S_ = 0.227, *p* < 0.02) in the *Shared focus* condition (see Additional file 10: Figure S2). Additional file 1: Table S1 shows correlations between % looking time for different ROIs and ASD symptoms severity as captured by the collected behavior rating scales.

## Discussion

We employed a dynamic activity monitoring paradigm in a sample of children and adults to quantify differences in social attention allocation between those with ASD and TD. We included two conditions in which actors either gazed at each other, or where their focus was on the activity, in order to determine whether these differences modulated visual attention. Individuals with ASD demonstrated different patterns of social attention during activity monitoring. Compared to the TD group, individuals with autism looked less at the actors’ heads, and longer at the shared activity area.

Contrary to expectations, we found that participants with autism looked more at the activity compared to participants without autism, but only in the *Mutual gaze* condition. There are several reasons why this may have been the case. First, as indicated by Shic et al. [27, 28], older individuals with ASD may not exhibit diminished activity monitoring to the same extent as 2-year-old toddlers with ASD, suggesting developmental changes in the monitoring of joint activities. The current study extends these findings by offering evidence that this upward developmental trajectory in ASD may continue as children grow older, ultimately reversing the pattern of diminished activity monitoring observed in younger children to that of a pattern of increased activity monitoring by school-age. Second, participants with ASD may focus more on activity due to increased preference toward areas of motion (i.e., hands manipulating the activity). Finally, this difference may be explained by how participants modulate their attention in response to differences in gaze behavior of the actors [31]. Participants with ASD did not adjust their attention to the activity based on where the actors were looking. That is, they spent the same amount of time attending to the activity during the *Mutual gaze* and *Shared focus* conditions. It is possible that the actor’s gaze direction may not have been salient to them and thus did not influence their looking behavior. In contrast, TD participants modulated attention such that in the *Mutual gaze* condition (relative to the *Shared focus* condition), TD participants spent more time looking at the actors’ heads and less time looking at the activity. Decreased responsiveness to gaze cues is in line with early joint attention deficits observed in toddlers with ASD and suggests that this difference persists across the lifespan, consistent with Freeth et al. [51].

The current study has similarities to a recent social attention study in adults, where richness of a social scene increased observable differences between ASD and TD groups in viewing of naturalistic videos [52]. These authors suggested that the ASD group did not pick up on the subtleties of increased social content (i.e., magnitude and quality of social content). Unlike the naturalistic and dynamic gaze shifts in that study, the gaze manipulations inherent within our activity monitoring stimuli intentionally disrupt the normative social modulation of gaze by utilizing fixed rather than dynamic gaze. It is therefore possible that socially savvy TD participants find this unnatural gaze modulation novel and eerily devoid of joint attention, driving their increased attention to the heads of the actors. Future work would benefit from understanding the extent by which group differences within this artificial manipulation of fixed gaze vary from natural interactional gaze patterns.

### Effects of age

In our sample of children and adults, age was found to have a significant effect on looking to the shared activity and to the heads of actors in the *Mutual gaze* condition, with older individuals with and without ASD looking less at the shared activity and more at the heads. Contrary to the studies in toddlers [24, 27, 28], individuals with ASD in our sample spent more time looking at the shared activity than TD controls. This may be consistent with developmental shifts in social interactions. In early childhood, social interaction is dominated by play and shared activities with objects. As children age, language and dyadic interactions become the primary mode of social interaction and learning, and more subtle non-verbal behaviors help to influence contextual interpretation of social behavior. Thus, the trends we see here may map on to typical developmental shifts in social interactions, where the most important information during a social interaction in adolescence and beyond is gleaned from language rather than a shared activity.

Of particular interest, there were no age-related group differences in activity monitoring, suggesting, based on Shic et al. [27, 28], that much of the differential developmental trajectory may be captured at the toddler age. It also suggests that, in terms of observed relationships between looking patterns and age, both TD and ASD groups change in a similar fashion over time. In addition, unlike in the toddler study, we did not find any differences between groups in visual attention to the background. Both groups payed more attention to the actors or the activity and the background “distractors” did not hold additional salience for the ASD group, as may have been predicted. This may be explained by similar developmental trajectories after the toddler age, whereby either biological motion, or motion is more salient than static objects. It could also be that the images used in the background were of less interest to older participants with ASD than the toddler group. In addition, unlike the toddler study, our current study did not include individuals with more severe intellectual impairment. Further study with manipulation of variables in older individuals with ASD, as well as with individuals with greater intellectual impairment, would be needed to determine the specific reasons for reduced attention to the background in an older ASD group.

### Understanding aspects of heterogeneity in ASD

Considering the established heterogeneity present in ASD, it is critical to understand how gaze patterns relate to prevalent individual differences, including sex, ASD symptomology, and cognitive abilities. First, unlike age, sex did not significantly contribute to any of the linear mixed-effects models, suggesting that while it may be important to account for this variable as a covariate [53], there is little evidence within the current study to suggest that patterns of social attention are influenced by sex. This finding is consistent with several studies that have not identified sex differences in behavioral features of ASD [54], but is in contrast to one study that identified sex differences in visual attention to dynamic social scenes for children with ASD [55]. Methodological differences between our study and Harrop et al. [55] could reconcile this discrepancy as the study included a younger, more restricted age range of participants between 6 and 10 years. It is possible that sex differences in social attention are developmentally sensitive, and that combining children, adolescents, and adults together may mask some developmental trends, and our sample size of 29 females compared to a larger proportion of males was not sufficient to detect any differences. Still, very few studies have examined sex differences in social attention for individuals with ASD across the lifespan, and our findings warrant further investigation.

We found that children rated as having more social affect challenges exhibited less attention to heads during the mutual gaze condition. In contrast, increased repetitive behaviors (particularly compulsive, ritualistic, sameness, and self-injurious behaviors) were related to increased attention to heads during the shared focus condition. These findings encompass both observations of child’s ASD symptoms (e.g., ADOS-2 total) and parental report (e.g., ABI core, RBS-R total) and are consistent with prior work indicating that looking behaviors correspond to social function and social behaviors in school-age children and adults with ASD [2, 56]. Within the current data, the lack of correspondence between social attention and social behaviors (e.g., SRS-2 or ABI social communication subdomain) is surprising. One possibility is that features of social attention are subtle and thus not well captured by macro-level parental report measures. However, other literature in infant populations has also not found reliable relationships between social attention and ASD symptoms [57–59] suggesting that perhaps heterogeneity may dilute the power to detect relationships at an individual level. Given the multiple comparisons that were made between eye-tracking features and behavior rating scales and the fact that the current study was not designed to thoroughly test relationships between severity of ASD symptoms and looking time, the few correlations described above should be treated with a great caution. The correlations can also be used to inform future research about the existence of potential links between behavioral reports and eye-tracking measures. Continuing to explore and replicate these findings with additional cohorts will be valuable in understanding how underlying ASD symptoms relate to implicit social attention patterns.

Our findings indicated that individuals with ASD with a higher IQ appear to look less at heads and bodies specifically in the context in which shared focus was on the activity, suggesting that individuals with higher cognitive ability direct more attention to the focus of the actors’ gaze. This finding suggests caution regarding the specificity of head-looking across the heterogeneity of ASD and is consistent with other recent work demonstrating that children with ASD who are minimally verbal are less likely to follow gaze shifts relative to age-matched verbal children with ASD within a spontaneous looking task [60]. One possibility is that children with a higher IQ are more likely to share focus with other people, unlike children with lower IQ, which may ultimately impact opportunities for implicit social learning. This may be because ASD children with a higher IQ are capable of processing the information quickly and are subsequently focused on the relevant scene content (i.e., direction of the actors’ gaze). Alternatively, it may be the case that monitoring activities is more related to mental age than relative IQ, and as such increased monitoring of activities in school-age children and adults with ASD may reflect cumulative effects of an atypical developmental progression. This is consistent with evidence indicating that toddlers and young children with higher developmental ability show increased monitoring of activities and diminished looking at the background [19, 27, 28].

#### Limitations

The current work has a number of limitations as we continue to examine possible use of activity monitoring as a biomarker. Firstly, population characteristics will need to be expanded for both groups of participants with and without autism in order to parse heterogeneity in ASD. For instance, while this study focused on a large, well-characterized sample of participants with ASD, similar efforts should be taken to characterize TD individuals to understand basic individual variability in activity monitoring. Further, biomarkers should be established and validated within individuals across development (i.e., longitudinal assessment and test–retest validity), as well as with participants with lower cognitive and/or adaptive functioning. Our sample was restricted by an inclusion criterion regarding cognitive ability (IQ > 60), which precluded our ability to evaluate individuals with more substantive intellectual disability. There remains a gap in the literature regarding how the activity monitoring paradigm, and social attention paradigms, in general, function in older and less cognitively able groups [21]. Associations between social attention and ASD symptomology should also be interpreted with caution due to the number of comparisons that were made and the possibility for spurious significant findings. Lastly, replication of this existing paradigm is required, including consistent methodology and analytics, as well as a better understanding of how activity monitoring gaze patterns respond to change (i.e., related to development or a specific treatment intervention).

## Conclusion

A key motivation for this work was to examine the potential of looking patterns during viewing of scenes depicting interactive activities to serve as a biomarker for ASD. Important features of a biomarker include robust differences between the clinical population and control group and persistent discriminative value throughout development, from infancy to adulthood. To this end, together with other current work [27, 28], we show that diminished looking to heads in certain contexts (i.e., mutual gaze) constitutes a potentially robust signature of ASD across the lifespan. By comparison, this same body of work suggests that looking at activities, while being a powerful predictor in very early childhood, may not have a strong discriminative ability later in childhood and adulthood. Identification of endophenotypic constructs may be more achievable in studies of infants, where skills are just emerging, but are likely to become more difficult and complex in older children and adults when interactions between life experiences, treatment effects, and compensatory mechanisms may play a role [2]. Social attention deficits at different ages may also vary along the developmental trajectory of ASD. Finally, there may be a distinction between diagnostic and phenotypic biomarkers, such that some tasks are more sensitive to the potentially binary diagnostic classification of ASD, whereas other tasks are more sensitive to the phenotypic heterogeneity observed *within* ASD [9].

We currently represent collaborations across multiple institutions [1, 61] that seek to develop biomarkers for ASD. Continued support across research institutions will be necessary to better understand and validate eye tracking and other candidate biomarkers.

## Supplementary information

Additional file 1 Table S1. Correlations between % looking time for different ROIs and symptoms severity in participants with ASD. The data are presented for each of the two stimulus conditions separately. Cells contain Spearman partial correlation coefficients along with the corresponding two-sided *p* values in parentheses. The correlation coefficients are computed on the data of all participants with ASD, with participant’s age, sex and KBIT-2 intelligence composite score being used as covariates. Cells with *p* values below 0.05 are highlighted in bold. n indicates the number of participants with the data of the corresponding behavior rating scale available. *ASD* autism spectrum disorder, *KBIT-2* Kaufmann Brief Intelligence Test-2, *ROI* region-of-interest

Additional file 2 Table S2. Fixed effects in linear mixed-effects models of different ROIs. Significance of the fixed effects is assessed using analysis of variance type III sum of squares and the Wald *χ*^2^ test. *p* values below 0.05 are highlighted in bold. *df* degrees of freedom, *ROI* region-of-interest.

Additional file 3 Table S3. Least-squares mean estimates, standard errors and two-sided 95% confidence intervals for different levels of the modelled categorical factors. *ASD* autism spectrum disorder, *df* degrees of freedom, *ROI* region-of-interest, *TD* typically developing.

Additional file 4 Table S4. Pair-wise comparisons of % looking time between the two groups of participants and stimulus conditions. Post-hoc pair-wise comparisons are performed using the Tukey–Kramer correction for multiple comparisons. *p* values below 0.05 are highlighted in bold. Cohen’s d is computed using all available data without selecting the same participants across stimulus conditions (see Additional file 8: Table S8). *ASD* autism spectrum disorder, *df* degrees of freedom, *ROI* region-of-interest, *SE* standard error, *TD* typically developing

Additional file 5 Table S5. Coefficients of determination *R*^2^ in linear mixed-effects models of different ROIs. Marginal *R*^2^ corresponds to the proportion of the total variance explained by the fixed effects, whereas conditional *R*^2^ is the proportion of the variance explained by both fixed and random effects [62]. The same models as that in Additional file 2: Table S2 are analyzed. *ROI* region-of-interest.

Additional file 6 Table S6. Coefficients of determination *R*^2^ in linear mixed-effects models comparing slopes of the relationships between participant’s age and % looking time across the two groups of participants. Marginal *R*^2^ corresponds to the proportion of the total variance explained by the fixed effects, whereas conditional *R*^2^ is the proportion of the variance explained by both fixed and random effects [62]. The same models as those in Additional file 7: Table S7 are analyzed. *ROI* region-of-interest.

Additional file 7 Table S7. Fixed effects in linear mixed-effects models comparing slopes of the relationships between participants’ age and % looking time across the two groups of participants. Significance of the fixed effects is assessed using analysis of variance type III sum of squares and the Wald *χ*^2^ test. *p* values below 0.05 are highlighted in bold. *df* degrees of freedom, *ROI* region-of-interest.

Additional file 8 Table S8. Participant characteristics for each stimulus condition separately. *n* indicates the number of participants. *ASD* autism spectrum disorder, *ADOS-2* Autism Diagnostic Observation Schedule, 2nd Edition, *KBIT-2* Kaufmann Brief Intelligence Test-2, *TD* typically developing.

Additional file 9 Figure S1. Scatter plots between the KBIT-2 IQ composite score and looking time for the ROIs *Bodies* and *Heads.* Only the data of individuals with ASD are presented. The data are presented for each stimulus condition (**a**, **c**—*Shared focus*; **b**, **d**—*Mutual gaze*) and ROI (**a**, **b**—*Bodies*; **c**, **d**—*Heads*) separately. Red dots denote individual participants, with n indicating their total number. The red line in each panel represents the best linear fit of the presented data. *r*_S_ in each panel correspond to a Spearman partial correlation coefficient computed on the data presented in that panel, with the corresponding *p* value being shown in parentheses. *ASD* autism spectrum disorder, *KBIT-2* Kaufmann Brief Intelligence Test-2.

Additional file 10 Figure S2. Scatter plots of significant relationships between looking time and the total score of behavior rating scales. Information about the significant relationships and their strength is provided in Table 2. The *x*- and *y*-axes of each panel correspond to the total score of a behavior rating scale and looking time for a specific ROI, respectively. The panel title reports the ROI and stimulus condition in which statistical significance is obtained. *r*_S_ in each panel correspond to a Spearman partial correlation coefficient computed on the data presented in that panel, with the corresponding *p* value being shown in parentheses. Red dots denote individual participants, with n indicating their number. The red line in each panel represents the best linear fit of the presented data. *ABI* Autism Behavior Inventory, *ADOS-2* Autism Diagnostic Observation Schedule, 2nd Edition, *RBS-R* Repetitive Behavior Scale—Revised, *ROI* region-of-interest.

Additional file 11 Figure S3. Between-group comparisons of the level of visual attention to presented stimuli. The data are presented for each of the two stimulus conditions separately. The red and blue colors correspond to the data of individuals with ASD and TD controls, respectively. The arrows along the *x*-axis indicate medians of the corresponding histograms matched by color. *n* is the number of participants. Bin width is 5%. *ASD* autism spectrum disorder, *TD* typically developing.

Additional file 12 Table S9. Fixed effects in the linear mixed-effects model that includes ROI and all its interactions with stimulus condition and participant group. To account for correlations between % looking time for different ROIs, the tested model utilizes the data of all ROIs, except for the ROI *Background*. Significance of the fixed effects is assessed using analysis of variance type III sum of squares and the Wald *χ*^2^ test. *p* values below 0.05 are highlighted in bold. *df* degrees of freedom, *ROI* region-of-interest.

Additional file 13 Table S10. Least-squares mean estimates, standard errors and two-sided 95% confidence intervals for different levels of the categorical factors in the linear mixed-effects model that includes ROI and all its interactions with stimulus condition and participant group. The tested model is the same as that presented in Additional file 12: Table S9. *ASD* autism spectrum disorder, *df* degrees of freedom, *ROI* region-of-interest, *TD* typically developing

Additional file 14 Table S11. Pair-wise comparisons of % looking time between the two groups of participants and stimulus conditions for each ROI separately in the linear mixed-effects model that includes ROI and all its interactions with stimulus condition and participant group. The tested model is the same as that presented in Additional file 12: Table S9 and Additional file 13: Table S10. Post-hoc pair-wise comparisons are performed using the Tukey–Kramer correction for multiple comparisons. The table presents only the results of comparisons within a single ROI but not between any two different ROIs. *p* values below 0.05 are highlighted in bold. Cohen’s d is computed using all available data without selecting the same participants across stimulus conditions (see Additional file 8: Table S8). *ASD* autism spectrum disorder, *df* degrees of freedom, *ROI* region-of-interest, *SE* standard error, *TD* typically developing

Additional file 15 Figure S4. Distributions of % looking time for each individual ROI, stimulus condition and participant group in the linear mixed-effects model that includes ROI and all its interactions with the latter two factors. The data of each ROI, stimulus condition and group of participants (ASD—red, TD—blue) are summarized in a form of boxplots. Black dots denote individual participants. n indicates the number of participants. * p value < 0.05, ** *p* value < 0.005, *** *p* value < 0.0005 (corrected for multiple comparisons for each individual ROI using the Tukey–Kramer method; see Additional file 13: Table S10 and Additional file 14: Table S11). Note that the model does not include data of the ROI *Background* (see Additional file 12: Table S9). *ASD* autism spectrum disorder, *ROI* region-of-interest, *TD* typically developing.

Additional file 16 Table S12. Fixed effects in the linear mixed-effects models that compare slopes of the relationships between participant’s age and % looking time between the two groups of participants while accounting for the effect of stimulus condition. The tested models are similar to those presented in Additional file 7: Table S7 but include stimulus condition and all its interactions with participant’s age and group as additional fixed effects. Significance of the fixed effects is assessed using analysis of variance type III sum of squares and the Wald *χ*^2^ test. *p* values below 0.05 are highlighted in bold. *df* degrees of freedom, *ROI* region-of-interest

Additional file 17 Table S13. Fixed effects in the linear mixed-effect model that compare slopes of the relationships between participant’s age and % looking time between the two groups of participants while accounting for the effects of stimulus condition and region of interest. The tested model is similar to those presented in Additional file 7: Table S7 and Additional file 16: Table S12 but includes stimulus condition and region of interest as well as all interactions between the latter two factors and participant’s age and group as additional fixed effects. Only data of the ROIs *Activity* and *Heads* are analyzed. Significance of the fixed effects is assessed using analysis of variance type III sum of squares and the Wald *χ*^2^ test. *p* values below 0.05 are highlighted in bold. *df* degrees of freedom, *ROI* region-of-interest.

Additional file 18 Table S14. Relationship between participant’s age and % looking time for the ROIs *Activity* and *Heads* tested for the groups of participants below a specified age. Only data of the participants below a specified age are analyzed, with n indicating their total number for each of the two stimulus conditions and groups of participants separately. *r*_S_ corresponds to a Spearman partial correlation coefficient computed on the data of both groups of participants for each selection of participants and stimulus condition separately. The corresponding two-sided *p* value is shown in parentheses. CI corresponds to a 95% equal-tailed two-sided confidence interval for the computed correlation coefficient. *p* values below 0.05 and confidence intervals that do not include 0 are highlighted in bold. *ASD* autism spectrum disorder, *CI* confidence interval, *ROI* region-of-interest, *TD* typically developing

Additional file 19: Table S15. Fixed effects in linear mixed-effects models comparing slopes of the relationships between participant’s age and % looking time across the two groups of participants that are below a specified age. Significance of the fixed effects is assessed using analysis of variance type III sum of squares and the Wald *χ*^2^ test. *p* values below 0.05 are highlighted in bold. *df* degrees of freedom, *ROI* region-of-interest.

Additional file 20 Table S16. Mean % looking time for each individual region of interest, stimulus condition, group of participants and clinical site. *n* indicates the number of analyzed participants. *ASD* autism spectrum disorder, *TD* typically developing.

## Data Availability

All data generated or analyzed during this study are included in this published article [and its supplementary information files].

## Abbreviations

ABC: Aberrant Behavior Checklist—Community
ABI: Autism Behavior Inventory
ADOS-2: Autism Diagnostic Observation Schedule, 2nd Edition
ASD: Autism spectrum disorder
CASI: Child Adolescent Symptom Inventory
CASI-Anx: Child Adolescent Symptom Inventory—Anxiety
*df*: Degrees of freedom
IQ: Intelligence quotient
KBIT-2: Kaufmann Brief Intelligence Test-2
RBS-R: Repetitive Behavior Scale—Revised
ROI: Region-of-interest
rs: Reported correlations
SD: Standard deviation
SE: Standard error
SRS-2: Social Responsiveness Scale 2™
TD: Typically developing

## Previous presentations

Data from this study were submitted as an abstract and accepted for presentation at the 32^nd^ ECNP Congress, September 8, 2019, Copenhagen, Denmark.
