# Supplementary material for: Social attention to activities in children and adults with autism spectrum disorder: effects of context and age
Source: Mol Autism. 2020 Oct 19;11:79. doi: 10.1186/s13229-020-00388-5 (PMC7574440; doi:10.1186/s13229-020-00388-5)
Supplement: Supplementary file 1 — Table S1. Correlations between % looking time for different ROIs and symptoms severity in participants with ASD. The data are presented for each of the two stimulus conditions separately. Cells contain Spearman partial correlation coefficients along with the corresponding two-sided p values in parentheses. The correlation coefficients are computed on the data of all participants with ASD, with participant’s age, sex and KBIT-2 intelligence composite score being used as covariates. Cells with p values below 0.05 are highlighted in bold. n indicates the number of participants with the data of the corresponding behavior rating scale available. ASD autism spectrum disorder, KBIT-2 Kaufmann Brief Intelligence Test-2, ROI region-of-interest [file 13229_2020_388_MOESM1_ESM.docx]

**Table S1.** Correlations between % looking time for different ROIs and symptoms severity in participants with autism.

| Behavior rating scale  ASD symptom | ROI | | Activity | Background | Bodies | Heads |
| --- | --- | --- | --- | --- | --- | --- |
|  | Condition | n |  |  |  |  |
| Autism Behavior Inventory | | |  |  |  |  |
| Core ASD symptom scale score | Shared focus | 120 | -0.090 (0.33) | 0.116 (0.21) | -0.007 (0.94) | 0.079 (0.40) |
|  | Mutual gaze | 107 | **-0.204 (0.04)** | 0.031 (0.75) | 0.042 (0.67) | 0.099 (0.32) |
| Challenging behavior | Shared focus | 120 | -0.118 (0.21) | 0.057 (0.54) | 0.068 (046) | 0.091 (0.33) |
|  | Mutual gaze | 107 | -0.161 (0.10) | 0.034 (0.74) | **0.195 (0.05)** | 0.069 (0.48) |
| Mental health | Shared focus | 120 | -0.144 (0.12) | 0.084 (0.37) | 0.032 (0.73) | 0.160 (0.09) |
|  | Mutual gaze | 107 | **-0.194 (0.05)** | -0.007 (0.94) | 0.060 (0.55) | 0.148 (0.13) |
| Restrictive repetitive behaviors | Shared focus | 120 | -0.123 (0.19) | 0.086 (0.36) | -0.030 (0.75) | 0.169 (0.07) |
|  | Mutual gaze | 107 | **-0.209 (0.04)** | -0.020 (0.84) | 0.030 (0.77) | 0.157 (0.11) |
| Self-regulation | Shared focus | 120 | -0.082 (0.38) | 0.081 (0.39) | 0.042 (0.65) | 0.119 (0.20) |
|  | Mutual gaze | 107 | -0.043 (0.67) | 0.027 (0.78) | 0.031 (0.75) | 0.030 (0.76) |
| Social communication | Shared focus | 120 | -0.023 (0.80) | 0.101 (0.28) | 0.019 (0.84) | -0.046 (0.62) |
|  | Mutual gaze | 107 | -0.102 (0.30) | 0.079 (0.43) | 0.034 (0.73) | -0.018 (0.86) |
| Autism Diagnostic Observation Schedule, 2nd edition | | |  |  |  |  |
| Restricted and repetitive behavior | Shared focus | 120 | 0.031 (0.74) | 0.069 (0.46) | -0.007 (0.94) | -0.112 (0.23) |
|  | Mutual gaze | 107 | 0.150 (0.13) | -0.003 (0.98) | -0.055 (0.58) | -0.106 (0.28) |

| Behavior rating scale  ASD symptom | ROI | | Activity | Background | Bodies | Heads |
| --- | --- | --- | --- | --- | --- | --- |
|  | Condition | n |  |  |  |  |
| Social affect | Shared focus | 120 | 0.049 (0.60) | 0.089 (0.34) | -0.023 (0.81) | -0.081 (0.38) |
|  | Mutual gaze | 107 | 0.094 (0.34) | 0.016 (0.87) | 0.035 (0.73) | **-0.243 (0.02)** |
| Total score | Shared focus | 120 | 0.030 (0.75) | 0.107 (0.25) | -0.040 (0.67) | -0.060 (0.52) |
|  | Mutual gaze | 107 | 0.097 (0.33) | 0.029 (0.77) | 0.015 (0.88) | **-0.212 (0.03)** |
| Aberrant Behavior Checklist | | |  |  |  |  |
| Hyperactivity non-compliance | Shared focus | 120 | -0.006 (0.95) | -0.043 (0.64) | 0.069 (0.46) | 0.110 (0.24) |
|  | Mutual gaze | 107 | -0.093 (0.35) | 0.020 (0.84) | 0.081 (0.41) | 0.067 (0.50) |
| Inappropriate speech | Shared focus | 120 | 0.015 (0.87) | -0.020 (0.83) | -0.033 (0.72) | 0.097 (0.30) |
|  | Mutual gaze | 107 | -0.077 (0.44) | -0.015 (0.88) | -0.027 (0.78) | 0.064 (0.52) |
| Irritability | Shared focus | 120 | -0.109 (0.24) | 0.045 (0.63) | 0.059 (0.52) | 0.139 (0.13) |
|  | Mutual gaze | 107 | **-0.194 (0.05)** | 0.097 (0.33) | 0.114 (0.25) | 0.074 (0.46) |
| Lethargy social withdrawal | Shared focus | 120 | 0.087 (0.35) | 0.014 (0.89) | 0.054 (0.56) | -0.052 (0.58) |
|  | Mutual gaze | 107 | -0.004 (0.96) | -0.042 (0.68) | 0.150 (0.13) | -0.092 (0.35) |
| Stereotypic behavior | Shared focus | 120 | 0.015 (0.87) | 0.095 (0.31) | 0.029 (0.76) | -0.087 (0.35) |
|  | Mutual gaze | 107 | 0.012 (0.90) | 0.014 (0.89) | 0.022 (0.82) | -0.057 (0.57) |
| Child Adolescent Symptom Inventory – Anxiety | | |  |  |  |  |
| Total score | Shared focus | 120 | -0.083 (0.37) | 0.051 (0.58) | -0.017 (0.86) | 0.151 (0.11) |
|  | Mutual gaze | 107 | -0.169 (0.09) | 0.005 (0.96) | -0.009 (0.93) | 0.100 (0.31) |

| Behavior rating scale  ASD symptom | ROI | | Activity | Background | Bodies | Heads |
| --- | --- | --- | --- | --- | --- | --- |
|  | Condition | n |  |  |  |  |
| Repetitive Behavior Scale – Revised | | |  |  |  |  |
| Compulsive behavior | Shared focus | 120 | -0.081 (0.39) | 0.001 (0.99) | -0.134 (0.15) | **0.206 (0.03)** |
|  | Mutual gaze | 107 | -0.029 (0.77) | -0.144 (0.14) | -0.075 (0.45) | 0.082 (0.41) |
| Ritualistic behavior | Shared focus | 120 | -0.074 (0.43) | -0.028 (0.77) | -0.112 (0.23) | **0.242 (0.01)** |
|  | Mutual gaze | 107 | **-0.199 (0.05)** | -0.036 (0.72) | -0.089 (0.37) | 0.170 (0.08) |
| Restricted behavior | Shared focus | 120 | -0.086 (0.36) | 0.011 (0.90) | -0.152 (0.10) | 0.165 (0.07) |
|  | Mutual gaze | 107 | -0.053 (0.60) | -0.016 (0.87) | -0.054 (0.59) | 0.076 (0.44) |
| Sameness behavior | Shared focus | 120 | -0.068 (0.47) | 0.004 (0.96) | -0.101 (0.28) | **0.200 (0.04)** |
|  | Mutual gaze | 107 | -0.164 (0.10) | 0.050 (0.61) | -0.009 (0.93) | 0.089 (0.37) |
| Self-injurious behavior | Shared focus | 120 | 0.099 (0.29) | -0.124 (0.18) | **-0.238 (0.01)** | **0.199 (0.04)** |
|  | Mutual gaze | 107 | -0.018 (0.86) | -0.115 (0.25) | **-0.244 (0.02)** | **0.253 (0.01)** |
| Stereotyped behavior | Shared focus | 120 | -0.031 (0.74) | 0.103 (0.27) | -0.091 (0.33) | 0.014 (0.88) |
|  | Mutual gaze | 107 | -0.067 (0.50) | 0.178 (0.07) | 0.010 (0.92) | -0.062 (0.53) |
| Total score | Shared focus | 120 | -0.085 (0.36) | 0.003 (0.98) | -0.124 (0.18) | **0.227 (0.02)** |
|  | Mutual gaze | 107 | -0.169 (0.09) | 0.003 (0.98) | -0.057 (0.56) | 0.137 (0.17) |
| Social Responsiveness Scale 2 | | |  |  |  |  |
| Social awareness | Shared focus | 119 | 0.010 (0.92) | 0.133 (0.15) | -0.074 (0.43) | -0.074 (0.43) |
|  | Mutual gaze | 106 | -0.011 (0.91) | 0.057 (0.57) | -0.104 (0.30) | -0.034 (0.74) |

| Behavior rating scale  ASD symptom | ROI | | Activity | Background | Bodies | Heads |
| --- | --- | --- | --- | --- | --- | --- |
|  | Condition | n |  |  |  |  |
| Social cognition | Shared focus | 119 | -0.033 (0.73) | -0.034 (0.72) | -0.069 (0.46) | 0.152 (0.10) |
|  | Mutual gaze | 106 | -0.019 (0.85) | -0.094 (0.35) | 0.110 (0.27) | 0.143 (0.15) |
| Social communication | Shared focus | 119 | -0.020 (0.83) | 0.061 (0.52) | 0.016 (0.86) | 0.010 (0.92) |
|  | Mutual gaze | 106 | -0.131 (0.19) | 0.087 (0.38) | 0.057 (0.57) | -0.036 (0.72) |
| Social motivation | Shared focus | 119 | 0.025 (0.79) | -0.021 (0.82) | 0.061 (0.52) | -0.036 (0.70) |
|  | Mutual gaze | 106 | -0.080 (0.42) | 0.024 (0.81) | -0.040 (0.69) | 0.011 (0.91) |
| Restricted interests and repetitive behavior | Shared focus | 119 | -0.122 (0.19) | 0.107 (0.25) | -0.054 (0.56) | 0.094 (0.31) |
|  | Mutual gaze | 106 | -0.117 (0.24) | 0.002 (0.99) | -0.097 (0.33) | 0.093 (0.35) |
| Social communication and interaction | Shared focus | 119 | -0.023 (0.81) | 0.045 (0.63) | 0.006 (0.95) | 0.037 (0.69) |
|  | Mutual gaze | 106 | -0.107 (0.28) | 0.037 (0.71) | -0.010 (0.92) | 0.025 (0.80) |
| Total score | Shared focus | 119 | -0.059 (0.53) | 0.077 (0.41) | -0.021 (0.82) | 0.053 (0.57) |
|  | Mutual gaze | 106 | -0.123 (0.22) | 0.038 (0.70) | -0.048 (0.63) | 0.048 (0.63) |

The data are presented for each of the two stimulus conditions separately. Cells contain Spearman partial correlation coefficients along with the corresponding two-sided *p*-values in parentheses. The correlation coefficients are computed on the data of all participants with autism, with participant’s age, sex and KBIT-2 intelligence composite score being used as covariates. Cells with *p*-values below 0.05 are highlighted in bold. n indicates the number of participants with the data of the corresponding behavior rating scale available.

Abbreviations: ASD: autism spectrum disorder; KBIT-2: Kaufmann Brief Intelligence Test-2; ROI: region-of-interest.
