# Supplementary material for: Social attention to activities in children and adults with autism spectrum disorder: effects of context and age
Source: Mol Autism. 2020 Oct 19;11:79. doi: 10.1186/s13229-020-00388-5 (PMC7574440; doi:10.1186/s13229-020-00388-5)
Supplement: Supplementary file 2 — Table S2. Fixed effects in linear mixed-effects models of different ROIs. Significance of the fixed effects is assessed using analysis of variance type III sum of squares and the Wald χ2 test. p values below 0.05 are highlighted in bold. df degrees of freedom, ROI region-of-interest. [file 13229_2020_388_MOESM2_ESM.docx]

**Table S2.** Fixed effects in linear mixed-effects models of different ROIs.

| ROI | Fixed effect | χ^2^-statistic | df | *p*-value |
| --- | --- | --- | --- | --- |
| Activity | Intercept | 660.4167 | 1 | **< 0.0001** |
|  | Stimulus condition | 3.3872 | 1 | 0.065704 |
|  | Participant group | 9.1514 | 1 | **0.002485** |
|  | Participant’s age | 16.0532 | 1 | **< 0.0001** |
|  | Participant’s sex | 3.0838 | 1 | 0.079077 |
|  | Stimulus condition x Participant group | 4.2263 | 1 | **0.039803** |
| Background | Intercept | 85.1494 | 1 | **0.0001** |
|  | Stimulus condition | 0.9193 | 1 | 0.3377 |
|  | Participant group | 1.1897 | 1 | 0.2754 |
|  | Participant’s age | 0.0939 | 1 | 0.7593 |
|  | Participant’s sex | 1.6043 | 1 | 0.2053 |
|  | Stimulus condition x Participant group | 0.4465 | 1 | 0.5040 |
| Bodies | Intercept | 46.4419 | 1 | **< 0.0001** |
|  | Stimulus condition | 13.3438 | 1 | **0.0002593** |
|  | Participant group | 2.6199 | 1 | 0.1055327 |
|  | Participant’s age | 1.0857 | 1 | 0.2974309 |
|  | Participant’s sex | 0.4193 | 1 | 0.5172673 |
|  | Stimulus condition x Participant group | 0.7224 | 1 | 0.3953506 |
| Heads | Intercept | 24.6793 | 1 | **< 0.0001** |
|  | Stimulus condition | 0.3728 | 1 | 0.541470 |
|  | Participant group | 36.2535 | 1 | **< 0.0001** |
|  | Participant’s age | 17.5508 | 1 | **0.0001** |
|  | Participant’s sex | 0.3951 | 1 | 0.529647 |
|  | Stimulus condition x Participant group | 8.4466 | 1 | **0.003657** |

Significance of the fixed effects is assessed using analysis of variance type III sum of squares and the Wald χ^2^ test. *p*‑values below 0.05 are highlighted in bold.

Abbreviations: df: degrees of freedom; ROI: region-of-interest.
