# Supplementary material for: Social attention to activities in children and adults with autism spectrum disorder: effects of context and age
Source: Mol Autism. 2020 Oct 19;11:79. doi: 10.1186/s13229-020-00388-5 (PMC7574440; doi:10.1186/s13229-020-00388-5)
Supplement: Supplementary file 3 — Table S3. Least-squares mean estimates, standard errors and two-sided 95% confidence intervals for different levels of the modelled categorical factors. ASD autism spectrum disorder, df degrees of freedom, ROI region-of-interest, TD typically developing. [file 13229_2020_388_MOESM3_ESM.docx]

**Table S3.** Least-squares mean estimates, standard errors and two-sided 95% confidence intervals for different levels of the modelled categorical factors.

| ROI | Participant group | Stimulus condition | Least-squares mean | Standard error | df | 95% Confidence interval |
| --- | --- | --- | --- | --- | --- | --- |
| Activity | ASD | Mutual gaze | 57.5 | 1.27 | 158 | (55.0, 60.0) |
|  | TD | Mutual gaze | 50.6 | 1.98 | 158 | (46.7, 54.5) |
|  | ASD | Shared focus | 59.5 | 1.23 | 142 | (57.1, 62.0) |
|  | TD | Shared focus | 57.0 | 1.97 | 142 | (53.1, 60.9) |
| Background | ASD | Mutual gaze | 18.8 | 0.962 | 158 | (16.9, 20.7) |
|  | TD | Mutual gaze | 16.9 | 1.508 | 158 | (13.9, 19.9) |
|  | ASD | Shared focus | 18.0 | 0.931 | 142 | (16.1, 19.8) |
|  | TD | Shared focus | 14.9 | 1.495 | 142 | (12.0, 17.9) |
| Bodies | ASD | Mutual gaze | 8.69 | 0.560 | 158 | (7.59, 9.80) |
|  | TD | Mutual gaze | 7.06 | 0.878 | 158 | (5.32, 8.79) |
|  | ASD | Shared focus | 7.08 | 0.545 | 142 | (6.00, 8.16) |
|  | TD | Shared focus | 6.17 | 0.872 | 142 | (4.45, 7.90) |
| Heads | ASD | Mutual gaze | 15.0 | 0.964 | 158 | (13.1, 19.9) |
|  | TD | Mutual gaze | 25.4 | 1.512 | 158 | (22.5, 28.4) |
|  | ASD | Shared focus | 15.4 | 0.942 | 142 | (13.5, 17.3) |
|  | TD | Shared focus | 21.9 | 1.503 | 142 | (18.9, 24.9) |

Abbreviations: ASD: autism spectrum disorder; df: degrees of freedom; ROI: region-of-interest; TD: typically developing.
