# Supplementary material for: Social attention to activities in children and adults with autism spectrum disorder: effects of context and age
Source: Mol Autism. 2020 Oct 19;11:79. doi: 10.1186/s13229-020-00388-5 (PMC7574440; doi:10.1186/s13229-020-00388-5)
Supplement: Supplementary file 4 — Table S4. Pair-wise comparisons of % looking time between the two groups of participants and stimulus conditions. Post-hoc pair-wise comparisons are performed using the Tukey–Kramer correction for multiple comparisons. p values below 0.05 are highlighted in bold. Cohen’s d is computed using all available data without selecting the same participants across stimulus conditions (see Additional file 8: Table S8). ASD autism spectrum disorder, df degrees of freedom, ROI region-of-interest, SE standard error, TD typically developing [file 13229_2020_388_MOESM4_ESM.docx]

**Table S4.** Pair-wise comparisons of % looking time between the two groups of participants and stimulus conditions.

| Comparison | Estimate | SE | Cohen’s d | df | *t*-ratio | *p*-value |
| --- | --- | --- | --- | --- | --- | --- |
| ROI = Activity |  |  |  |  |  |  |
| ASD, Mutual gaze vs. TD, Mutual gaze | 6.918 | 2.29 | 0.60 | 158 | 3.025 | **0.0152** |
| ASD, Mutual gaze vs. ASD, Shared focus | -2.007 | 1.09 | 0.12 | 142 | -1.840 | 0.2589 |
| ASD, Mutual gaze vs. TD, Shared focus | 0.561 | 2.27 | 0.11 | 142 | 0.247 | 0.9947 |
| ASD, Shared focus vs. TD, Shared focus | 2.568 | 2.25 | 0.22 | 142 | 1.142 | 0.6645 |
| ASD, Shared focus vs. TD, Mutual gaze | -8.925 | 2.26 | 0.69 | 142 | -3.941 | **0.0007** |
| TD, Mutual gaze vs. TD, Shared focus | -6.357 | 1.81 | 0.51 | 142 | -3.506 | **0.0034** |
| ROI = Background |  |  |  |  |  |  |
| ASD, Mutual gaze vs. TD, Mutual gaze | 1.897 | 1.739 | 0.22 | 158 | 1.091 | 0.6957 |
| ASD, Mutual gaze vs. ASD, Shared focus | 0.833 | 0.869 | 0.07 | 142 | 0.959 | 0.7730 |
| ASD, Mutual gaze vs. TD, Shared focus | 3.858 | 1.727 | 0.43 | 142 | 2.234 | 0.1190 |
| ASD, Shared focus vs. TD, Shared focus | 3.024 | 1.709 | 0.32 | 142 | 1.770 | 0.2920 |
| ASD, Shared focus vs. TD, Mutual gaze | -1.063 | 1.721 | 0.13 | 142 | -0.618 | 0.9262 |
| TD, Mutual gaze vs. TD, Shared focus | 1.961 | 1.447 | 0.22 | 142 | 1.355 | 0.5294 |
| ROI = Bodies |  |  |  |  |  |  |
| ASD, Mutual gaze vs. TD, Mutual gaze | 1.6346 | 1.010 | 0.26 | 158 | 1.619 | 0.3711 |
| ASD, Mutual gaze vs. ASD, Shared focus | 1.6129 | 0.442 | 0.23 | 142 | 3.653 | **0.0020** |
| ASD, Mutual gaze vs. TD, Shared focus | 2.5206 | 1.004 | 0.41 | 142 | 2.510 | 0.0626 |
| ASD, Shared focus vs. TD, Shared focus | 0.9077 | 0.996 | 0.17 | 142 | 0.912 | 0.7986 |
| ASD, Shared focus vs. TD, Mutual gaze | -0.0217 | 1.001 | 0.01 | 142 | -0.022 | 1.0000 |
| TD, Mutual gaze vs. TD, Shared focus | 0.8860 | 0.733 | 0.24 | 142 | 1.209 | 0.6219 |
| ROI = Heads |  |  |  |  |  |  |
| ASD, Mutual gaze vs. TD, Mutual gaze | -10.460 | 1.737 | 1.05 | 158 | -6.021 | **0.0001** |
| ASD, Mutual gaze vs. ASD, Shared focus | -0.428 | 0.702 | 0.04 | 142 | -0.611 | 0.9286 |
| ASD, Mutual gaze vs. TD, Shared focus | -6.943 | 1.728 | 0.76 | 142 | -4.017 | **0.0005** |
| ASD, Shared focus vs. TD, Shared focus | -6.515 | 1.715 | 0.75 | 142 | -3.798 | **0.0012** |
| ASD, Shared focus vs. TD, Mutual gaze | 10.031 | 1.724 | 1.05 | 142 | 5.817 | **0.0001** |
| TD, Mutual gaze vs. TD, Shared focus | 3.517 | 1.162 | 0.33 | 142 | 3.026 | **0.0154** |

Post-hoc pair-wise comparisons are performed using the Tukey-Kramer correction for multiple comparisons. *p*-values below 0.05 are highlighted in bold. Cohen’s d is computed using all data available for the compared combinations of participant group x stimulus condition (see Additional File 8: Table S8).

Abbreviations: ASD: autism spectrum disorder; df: degrees of freedom; ROI: region-of-interest; SE: standard error; TD: typically developing.
