# Supplementary material for: Social attention to activities in children and adults with autism spectrum disorder: effects of context and age
Source: Mol Autism. 2020 Oct 19;11:79. doi: 10.1186/s13229-020-00388-5 (PMC7574440; doi:10.1186/s13229-020-00388-5)
Supplement: Supplementary file 5 — Table S5. Coefficients of determination R2 in linear mixed-effects models of different ROIs. Marginal R2 corresponds to the proportion of the total variance explained by the fixed effects, whereas conditional R2 is the proportion of the variance explained by both fixed and random effects [62]. The same models as that in Additional file 2: Table S2 are analyzed. ROI region-of-interest. [file 13229_2020_388_MOESM5_ESM.docx]

**Table S5.** Coefficients of determination R^2^ in linear mixed-effects models of different ROIs.

| ROI | Marginal R^2^ | Conditional R^2^ |
| --- | --- | --- |
| Activity | 0.12 | 0.62 |
| Background | 0.03 | 0.54 |
| Bodies | 0.03 | 0.65 |
| Heads | 0.22 | 0.76 |

Marginal R^2^ corresponds to the proportion of the total variance explained by the fixed effects, whereas conditional R^2^ is the proportion of the variance explained by both fixed and random effects^63^. The same models as that in Additional File 2: Table S2 are analyzed.

Abbreviations: ROI: region-of-interest.
