# Supplementary material for: Social attention to activities in children and adults with autism spectrum disorder: effects of context and age
Source: Mol Autism. 2020 Oct 19;11:79. doi: 10.1186/s13229-020-00388-5 (PMC7574440; doi:10.1186/s13229-020-00388-5)
Supplement: Supplementary file 6 — Table S6. Coefficients of determination R2 in linear mixed-effects models comparing slopes of the relationships between participant’s age and % looking time across the two groups of participants. Marginal R2 corresponds to the proportion of the total variance explained by the fixed effects, whereas conditional R2 is the proportion of the variance explained by both fixed and random effects [62]. The same models as those in Additional file 7: Table S7 are analyzed. ROI region-of-interest. [file 13229_2020_388_MOESM6_ESM.docx]

**Table S6.** Coefficients of determination R^2^ in linear mixed-effects models comparing slopes of the relationships between participant’s age and % looking time across the two groups of participants.

| ROI | Marginal R^2^ | Conditional R^2^ |
| --- | --- | --- |
| Activity | 0.09 | 0.58 |
| Heads | 0.21 | 0.75 |

Marginal R^2^ corresponds to the proportion of the total variance explained by the fixed effects, whereas conditional R^2^ is the proportion of the variance explained by both fixed and random effects^63^. The same models as those in Additional File 7: Table S7 are analyzed.

Abbreviations: ROI: region-of-interest.
