# Supplementary material for: Social attention to activities in children and adults with autism spectrum disorder: effects of context and age
Source: Mol Autism. 2020 Oct 19;11:79. doi: 10.1186/s13229-020-00388-5 (PMC7574440; doi:10.1186/s13229-020-00388-5)
Supplement: Supplementary file 7 — Table S7. Fixed effects in linear mixed-effects models comparing slopes of the relationships between participants’ age and % looking time across the two groups of participants. Significance of the fixed effects is assessed using analysis of variance type III sum of squares and the Wald χ2 test. p values below 0.05 are highlighted in bold. df degrees of freedom, ROI region-of-interest. [file 13229_2020_388_MOESM7_ESM.docx]

**Table S7.** Fixed effects in linear mixed-effects models comparing slopes of the relationships between participant’s age and % looking time across the two groups of participants.

| ROI | Fixed effect | χ^2^-statistic | df | *p*-value |
| --- | --- | --- | --- | --- |
| Activity | Intercept | 873.5531 | 1 | **< 0.0001** |
|  | Participant group | 0.5066 | 1 | 0.47661 |
|  | Participant’s age | 4.7377 | 1 | **0.02951** |
|  | Participant group x Participant’s age | 0.4893 | 1 | 0.48423 |
| Heads | Intercept | 51.6724 | 1 | **< 0.0001** |
|  | Participant group | 5.6645 | 1 | **0.01731** |
|  | Participant’s age | 5.9141 | 1 | **0.01502** |
|  | Participant group x Participant’s age | 0.6868 | 1 | 0.40727 |

Data for each ROI are pooled across the two stimulus conditions. Significance of the fixed effects is assessed using analysis of variance type III sum of squares and the Wald χ^2^ test. *p*‑values below 0.05 are highlighted in bold.

Abbreviations: df: degrees of freedom; ROI: region-of-interest.
