# Supplementary material for: Social attention to activities in children and adults with autism spectrum disorder: effects of context and age
Source: Mol Autism. 2020 Oct 19;11:79. doi: 10.1186/s13229-020-00388-5 (PMC7574440; doi:10.1186/s13229-020-00388-5)
Supplement: Supplementary file 8 — Table S8. Participant characteristics for each stimulus condition separately. n indicates the number of participants. ASD autism spectrum disorder, ADOS-2 Autism Diagnostic Observation Schedule, 2nd Edition, KBIT-2 Kaufmann Brief Intelligence Test-2, TD typically developing. [file 13229_2020_388_MOESM8_ESM.docx]

**Table S8.** Participant characteristics for each stimulus condition separately.

| Condition | | Shared focus | | Mutual gaze | |
| --- | --- | --- | --- | --- | --- |
| Characteristic | Group | ASD | TD | ASD | TD |
| Total number of participants, n | | 120 | 40 | 107 | 39 |
| Sex, n (%): | |  |  |  |  |
| Male | | 91 (75.8) | 26 (65.0) | 80 (74.8) | 25 (64.1) |
| Female | | 29 (24.2) | 14 (35.0) | 27 (25.2) | 14 (35.9) |
| χ^2^ test, *p*-value | | 0.16 | | 0.16 | |
| Age, years: | |  |  |  |  |
| Mean (SD) | | 14.6 (8.0) | 16.4 (13.3) | 14.6 (8.0) | 16.6 (13.4) |
| Median (range) | | 12.0 (6-54) | 11.5 (6-63) | 12.0 (6-54) | 12.0 (6-63) |
| Kolmogorov-Smirnov test, *p*-value | | 0.74 | | 0.76 | |
| ADOS-2 Total score, mean (SD, range) | | 7.6  (1.7, 4-10) | - | 7.6  (1.7, 4-10) | - |
| KBIT-2 IQ composite score, mean (SD, range) | | 98.9  (19.9, 60-136) | - | 98.4  (19.9, 60-136) | - |

n indicates the number of participants.

Abbreviations: ASD: autism spectrum disorder; ADOS-2: Autism Diagnostic Observation Schedule, 2nd edition; IQ: intelligence quotient; KBIT-2: Kaufmann Brief Intelligence Test-2; TD: typically developing.
