# Supplementary material for: Social attention to activities in children and adults with autism spectrum disorder: effects of context and age
Source: Mol Autism. 2020 Oct 19;11:79. doi: 10.1186/s13229-020-00388-5 (PMC7574440; doi:10.1186/s13229-020-00388-5)
Supplement: Supplementary file 9 — Figure S1. Scatter plots between the KBIT-2 IQ composite score and looking time for the ROIs Bodies and Heads. Only the data of individuals with ASD are presented. The data are presented for each stimulus condition (a, c—Shared focus; b, d—Mutual gaze) and ROI (a, b—Bodies; c, d—Heads) separately. Red dots denote individual participants, with n indicating their total number. The red line in each panel represents the best linear fit of the presented data. rS in each panel correspond to a Spearman partial correlation coefficient computed on the data presented in that panel, with the corresponding p value being shown in parentheses. ASD autism spectrum disorder, KBIT-2 Kaufmann Brief Intelligence Test-2. [file 13229_2020_388_MOESM9_ESM.docx]

**Figure S1.** Scatter plots between the KBIT-2 IQ composite score and looking time for the ROIs *Bodies* and *Heads*.

**
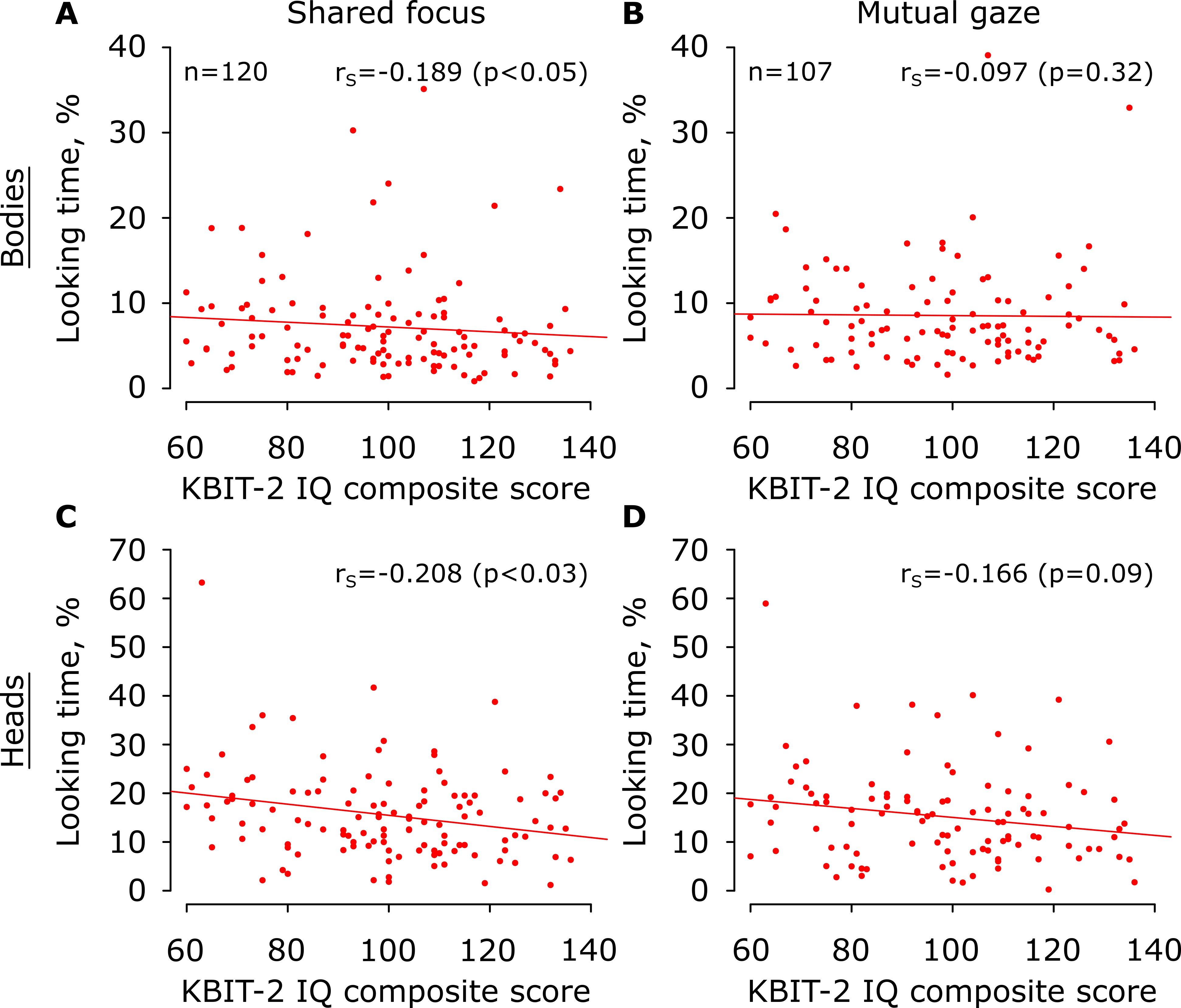
**

Only the data of individuals with ASD are presented. The data are presented for each stimulus condition (A, C – *Shared focus*; B, D – *Mutual gaze*) and ROI (A, B – *Bodies*; C, D – *Heads*) separately. Red dots denote individual participants, with n indicating their total number. The red line in each panel represents the best linear fit of the presented data. r_S_ in each panel correspond to a Spearman partial correlation coefficient computed on the data presented in that panel, with the corresponding *p*-value being shown in parentheses.

Abbreviations: ASD: autism spectrum disorder; KBIT-2: Kaufmann Brief Intelligence Test-2.
