# Supplementary material for: Social attention to activities in children and adults with autism spectrum disorder: effects of context and age
Source: Mol Autism. 2020 Oct 19;11:79. doi: 10.1186/s13229-020-00388-5 (PMC7574440; doi:10.1186/s13229-020-00388-5)
Supplement: Supplementary file 10 — Figure S2. Scatter plots of significant relationships between looking time and the total score of behavior rating scales. Information about the significant relationships and their strength is provided in Table 2. The x- and y-axes of each panel correspond to the total score of a behavior rating scale and looking time for a specific ROI, respectively. The panel title reports the ROI and stimulus condition in which statistical significance is obtained. rS in each panel correspond to a Spearman partial correlation coefficient computed on the data presented in that panel, with the corresponding p value being shown in parentheses. Red dots denote individual participants, with n indicating their number. The red line in each panel represents the best linear fit of the presented data. ABI Autism Behavior Inventory, ADOS-2 Autism Diagnostic Observation Schedule, 2nd Edition, RBS-R Repetitive Behavior Scale—Revised, ROI region-of-interest. [file 13229_2020_388_MOESM10_ESM.docx]

**Figure S2.** Scatter plots of significant relationships between looking time and the total score of behavior rating scales.


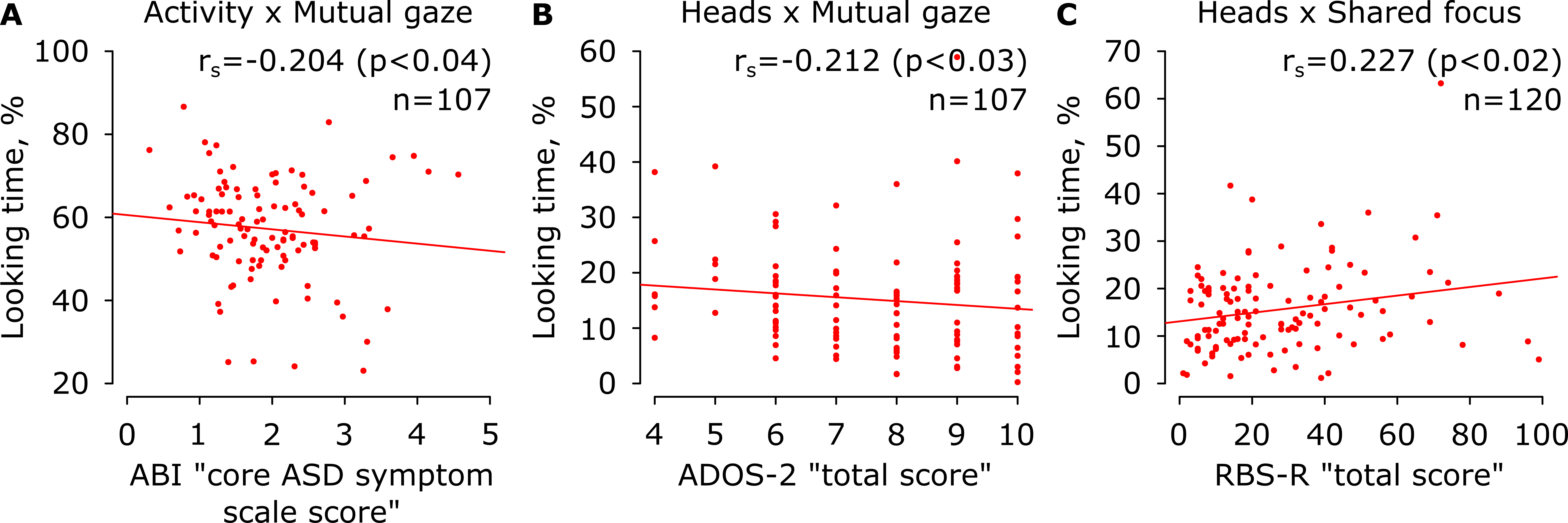


Information about the significant relationships and their strength is provided in Table 2. The x- and y-axes of each panel correspond to the total score of a behavior rating scale and looking time for a specific ROI, respectively. The panel title reports the ROI and stimulus condition in which statistical significance is obtained. r_S_ in each panel correspond to a Spearman partial correlation coefficient computed on the data presented in that panel, with the corresponding *p*-value being shown in parentheses. Red dots denote individual participants, with n indicating their number. The red line in each panel represents the best linear fit of the presented data.

Abbreviations: ABI: Autism Behavior Inventory; ADOS-2: Autism Diagnostic Observation Schedule, 2nd edition; RBS-R: Repetitive Behavior Scale – Revised; ROI: region-of-interest.
