# Supplementary material for: Social attention to activities in children and adults with autism spectrum disorder: effects of context and age
Source: Mol Autism. 2020 Oct 19;11:79. doi: 10.1186/s13229-020-00388-5 (PMC7574440; doi:10.1186/s13229-020-00388-5)
Supplement: Supplementary file 11 — Figure S3. Between-group comparisons of the level of visual attention to presented stimuli. The data are presented for each of the two stimulus conditions separately. The red and blue colors correspond to the data of individuals with ASD and TD controls, respectively. The arrows along the x-axis indicate medians of the corresponding histograms matched by color. n is the number of participants. Bin width is 5%. ASD autism spectrum disorder, TD typically developing. [file 13229_2020_388_MOESM11_ESM.docx]

**Figure S3.** Between-group comparisons of the level of visual attention to presented stimuli.


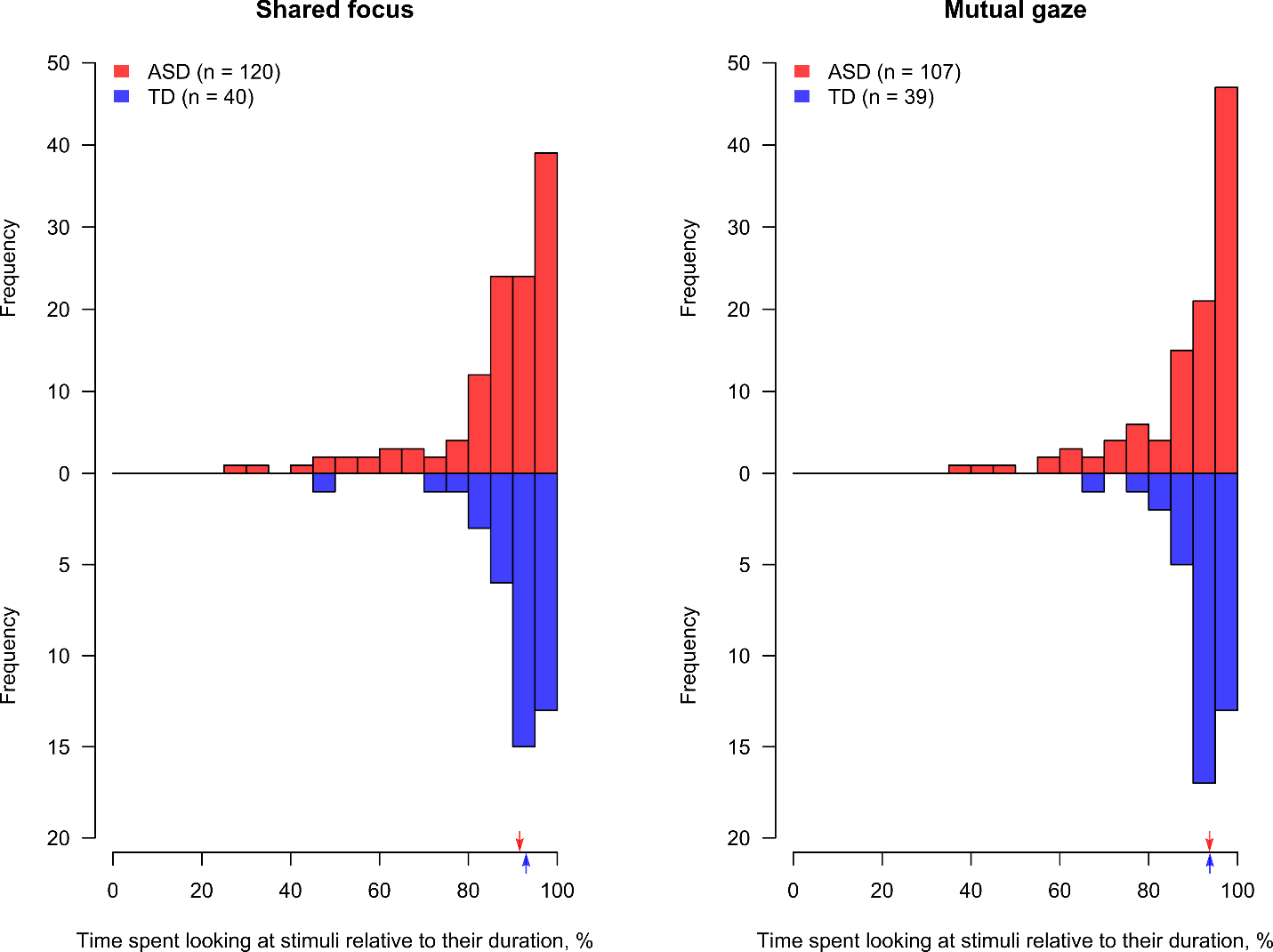


The data are presented for each of the two stimulus conditions separately. The red and blue colors correspond to the data of individuals with ASD and TD controls, respectively. The arrows along the x-axis indicate medians of the corresponding histograms matched by color. n is the number of participants. Bin width is 5%.

Abbreviations: ASD: autism spectrum disorder; TD: typically developing.
