# Supplementary material for: Social attention to activities in children and adults with autism spectrum disorder: effects of context and age
Source: Mol Autism. 2020 Oct 19;11:79. doi: 10.1186/s13229-020-00388-5 (PMC7574440; doi:10.1186/s13229-020-00388-5)
Supplement: Supplementary file 12 — Table S9. Fixed effects in the linear mixed-effects model that includes ROI and all its interactions with stimulus condition and participant group. To account for correlations between % looking time for different ROIs, the tested model utilizes the data of all ROIs, except for the ROI Background. Significance of the fixed effects is assessed using analysis of variance type III sum of squares and the Wald χ2 test. p values below 0.05 are highlighted in bold. df degrees of freedom, ROI region-of-interest. [file 13229_2020_388_MOESM12_ESM.docx]

**Table S9.** Fixed effects in the linear mixed-effects model that includes ROI and all its interactions with stimulus condition and participant group.

| Fixed effect | χ^2^-statistic | df | *p*-value |
| --- | --- | --- | --- |
| Intercept | 2113.4768 | 1 | **< 0.0001** |
| Stimulus condition | 1.4496 | 1 | 0.2286 |
| Participant group | 17.5992 | 1 | **< 0.0001** |
| Participant’s age | 0.0272 | 1 | 0.8689 |
| Participant’s sex | 0.5443 | 1 | 0.4607 |
| ROI | 1564.4175 | 2 | **< 0.0001** |
| Stimulus condition x Participant group | 3.5449 | 1 | 0.0597 |
| Stimulus condition x ROI | 2.4806 | 2 | 0.2893 |
| Participant group x ROI | 52.7439 | 2 | **< 0.0001** |
| Stimulus condition x Participant group x ROI | 5.8572 | 2 | 0.0535 |

To account for correlations between % looking time for different ROIs, the tested model utilizes the data of all ROIs, except for the ROI *Background*. Significance of the fixed effects is assessed using analysis of variance type III sum of squares and the Wald χ^2^ test. *p*‑values below 0.05 are highlighted in bold.

Abbreviations: df: degrees of freedom; ROI: region-of-interest.
