# Supplementary material for: Social attention to activities in children and adults with autism spectrum disorder: effects of context and age
Source: Mol Autism. 2020 Oct 19;11:79. doi: 10.1186/s13229-020-00388-5 (PMC7574440; doi:10.1186/s13229-020-00388-5)
Supplement: Supplementary file 13 — Table S10. Least-squares mean estimates, standard errors and two-sided 95% confidence intervals for different levels of the categorical factors in the linear mixed-effects model that includes ROI and all its interactions with stimulus condition and participant group. The tested model is the same as that presented in Additional file 12: Table S9. ASD autism spectrum disorder, df degrees of freedom, ROI region-of-interest, TD typically developing [file 13229_2020_388_MOESM13_ESM.docx]

**Table S10.** Least-squares mean estimates, standard errors and two-sided 95% confidence intervals for different levels of the categorical factors in the linear mixed-effects model that includes ROI and all its interactions with stimulus condition and participant group.

| ROI | Participant group | Stimulus condition | Least-squares mean | Standard error | df | 95% Confidence interval |
| --- | --- | --- | --- | --- | --- | --- |
| Activity | ASD | Mutual gaze | 57.3 | 0.96 | 158 | (55.4, 59.2) |
|  | TD | Mutual gaze | 49.7 | 1.57 | 158 | (46.6, 52.8) |
|  | ASD | Shared focus | 58.9 | 0.91 | 158 | (57.1, 60.7) |
|  | TD | Shared focus | 56.0 | 1.55 | 158 | (53.0, 59.1) |
| Bodies | ASD | Mutual gaze | 8.68 | 0.96 | 158 | (6.79, 10.6) |
|  | TD | Mutual gaze | 7.29 | 1.57 | 158 | (4.19, 10.4) |
|  | ASD | Shared focus | 7.37 | 0.91 | 158 | (5.58, 9.17) |
|  | TD | Shared focus | 6.41 | 1.55 | 158 | (3.35, 9.47) |
| Heads | ASD | Mutual gaze | 15.3 | 0.96 | 158 | (13.4, 17.2) |
|  | TD | Mutual gaze | 26.1 | 1.57 | 158 | (23.0, 29.2) |
|  | ASD | Shared focus | 15.7 | 0.91 | 158 | (13.9, 17.5) |
|  | TD | Shared focus | 22.6 | 1.55 | 158 | (19.5, 25.6) |

The tested model is the same as that presented in Additional File 12: Table S9.

Abbreviations: ASD: autism spectrum disorder; df: degrees of freedom; ROI: region-of-interest; TD: typically developing.
