# Supplementary material for: Social attention to activities in children and adults with autism spectrum disorder: effects of context and age
Source: Mol Autism. 2020 Oct 19;11:79. doi: 10.1186/s13229-020-00388-5 (PMC7574440; doi:10.1186/s13229-020-00388-5)
Supplement: Supplementary file 14 — Table S11. Pair-wise comparisons of % looking time between the two groups of participants and stimulus conditions for each ROI separately in the linear mixed-effects model that includes ROI and all its interactions with stimulus condition and participant group. The tested model is the same as that presented in Additional file 12: Table S9 and Additional file 13: Table S10. Post-hoc pair-wise comparisons are performed using the Tukey–Kramer correction for multiple comparisons. The table presents only the results of comparisons within a single ROI but not between any two different ROIs. p values below 0.05 are highlighted in bold. Cohen’s d is computed using all available data without selecting the same participants across stimulus conditions (see Additional file 8: Table S8). ASD autism spectrum disorder, df degrees of freedom, ROI region-of-interest, SE standard error, TD typically developing [file 13229_2020_388_MOESM14_ESM.docx]

**Table S11.** Pair-wise comparisons of % looking time between the two groups of participants and stimulus conditions for each ROI separately in the linear mixed-effects model that includes ROI and all its interactions with stimulus condition and participant group.

| Comparison | Estimate | SE | Cohen’s d | df | *t*-ratio | *p*-value |
| --- | --- | --- | --- | --- | --- | --- |
| ROI = Activity |  |  |  |  |  |  |
| ASD, Mutual gaze vs. TD, Mutual gaze | 7.666 | 1.83 | 0.60 | 158 | 4.195 | **0.0026** |
| ASD, Mutual gaze vs. ASD, Shared focus | -1.562 | 1.30 | 0.12 | 746 | -1.204 | 0.9886 |
| TD, Mutual gaze vs. TD, Shared focus | -6.364 | 2.20 | 0.51 | 746 | -2.898 | 0.1435 |
| ASD, Shared focus vs. TD, Shared focus | 2.864 | 1.78 | 0.22 | 158 | 1.606 | 0.9045 |
| ROI = Bodies |  |  |  |  |  |  |
| ASD, Mutual gaze vs. TD, Mutual gaze | 1.394 | 1.83 | 0.26 | 158 | 0.763 | 0.9998 |
| ASD, Mutual gaze vs. ASD, Shared focus | 1.309 | 1.30 | 0.23 | 746 | 1.009 | 0.9975 |
| TD, Mutual gaze vs. TD, Shared focus | 0.879 | 2.20 | 0.24 | 746 | 0.400 | 1.0000 |
| ASD, Shared focus vs. TD, Shared focus | 0.964 | 1.78 | 0.17 | 158 | 0.540 | 1.0000 |
| ROI = Heads |  |  |  |  |  |  |
| ASD, Mutual gaze vs. TD, Mutual gaze | -10.767 | 1.83 | 1.05 | 158 | -5.892 | **0.0001** |
| ASD, Mutual gaze vs. ASD, Shared focus | -0.411 | 1.30 | 0.04 | 746 | -0.317 | 1.0000 |
| TD, Mutual gaze vs. TD, Shared focus | 3.516 | 2.20 | 0.33 | 746 | 1.601 | 0.9085 |
| ASD, Shared focus vs. TD, Shared focus | -6.840 | 1.78 | 0.75 | 158 | -3.835 | **0.0096** |

The tested model is the same as that presented in Additional File 12: Table S9 and Additional File 13: Table S10. Post-hoc pair-wise comparisons are performed using the Tukey-Kramer correction for multiple comparisons. The table presents only the results of comparisons within a single ROI but not between any two different ROIs. *p*-values below 0.05 are highlighted in bold. Cohen’s d is computed using all data available for the compared combinations of participant group x stimulus condition (see Additional File 8: Table S8).

Abbreviations: ASD: autism spectrum disorder; df: degrees of freedom; ROI: region-of-interest; SE: standard error; TD: typically developing.
