# Supplementary material for: Social attention to activities in children and adults with autism spectrum disorder: effects of context and age
Source: Mol Autism. 2020 Oct 19;11:79. doi: 10.1186/s13229-020-00388-5 (PMC7574440; doi:10.1186/s13229-020-00388-5)
Supplement: Supplementary file 15 — Figure S4. Distributions of % looking time for each individual ROI, stimulus condition and participant group in the linear mixed-effects model that includes ROI and all its interactions with the latter two factors. The data of each ROI, stimulus condition and group of participants (ASD—red, TD—blue) are summarized in a form of boxplots. Black dots denote individual participants. n indicates the number of participants. * p value < 0.05, ** p value < 0.005, *** p value < 0.0005 (corrected for multiple comparisons for each individual ROI using the Tukey–Kramer method; see Additional file 13: Table S10 and Additional file 14: Table S11). Note that the model does not include data of the ROI Background (see Additional file 12: Table S9). ASD autism spectrum disorder, ROI region-of-interest, TD typically developing. [file 13229_2020_388_MOESM15_ESM.docx]

**Figure S4.** Distributions of % looking time for each individual ROI, stimulus condition and participant group in the linear mixed-effects model that includes ROI and all its interactions with the latter two factors.


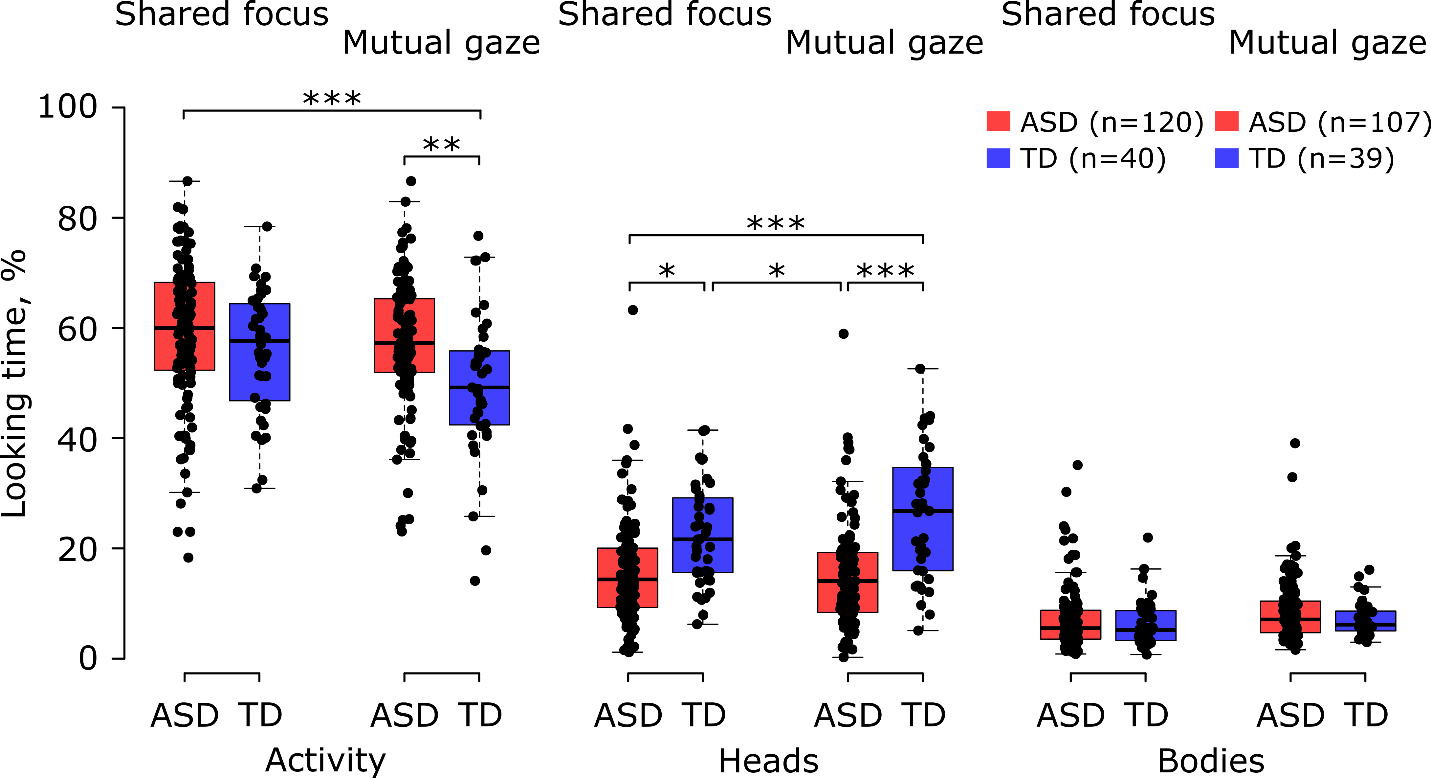


The data of each ROI, stimulus condition and group of participants (ASD – red, TD – blue) are summarized in a form of boxplots. Black dots denote individual participants. n indicates the number of participants. *** *p*-value < 0.05, ** *p*-value < 0.005, *** *p*-value < 0.0005 (corrected for multiple comparisons for each individual ROI using the Tukey-Kramer method; see Additional File 13: Table S10 and Additional File 14: Table S11). Note that the model does not include data of the ROI *Background* (see Additional File 12: Table S9).

Abbreviations: ASD: autism spectrum disorder; ROI: region-of-interest; TD: typically developing.
