# Supplementary material for: Social attention to activities in children and adults with autism spectrum disorder: effects of context and age
Source: Mol Autism. 2020 Oct 19;11:79. doi: 10.1186/s13229-020-00388-5 (PMC7574440; doi:10.1186/s13229-020-00388-5)
Supplement: Supplementary file 16 — Table S12. Fixed effects in the linear mixed-effects models that compare slopes of the relationships between participant’s age and % looking time between the two groups of participants while accounting for the effect of stimulus condition. The tested models are similar to those presented in Additional file 7: Table S7 but include stimulus condition and all its interactions with participant’s age and group as additional fixed effects. Significance of the fixed effects is assessed using analysis of variance type III sum of squares and the Wald χ2 test. p values below 0.05 are highlighted in bold. df degrees of freedom, ROI region-of-interest [file 13229_2020_388_MOESM16_ESM.docx]

**Table S12.** Fixed effects in the linear mixed-effects models that compare slopes of the relationships between participant’s age and % looking time between the two groups of participants while accounting for the effect of stimulus condition.

| ROI | Fixed effect | χ^2^-statistic | df | *p*-value |
| --- | --- | --- | --- | --- |
| Activity | Intercept | 701.8563 | 1 | < **0.0001** |
|  | Participant group | 1.5161 | 1 | 0.21821 |
|  | Participant’s age | 11.1458 | 1 | **0.00084** |
|  | Stimulus condition | 2.7102 | 1 | 0.09971 |
|  | Participant group x Participant’s age | 0.1725 | 1 | 0.67786 |
|  | Participant group x Stimulus condition | 1.4803 | 1 | 0.22372 |
|  | Participant’s age x Stimulus condition | 8.5419 | 1 | **0.00347** |
|  | Participant group x Participant’s age x Stimulus condition | 0.0524 | 1 | 0.81892 |
| Heads | Intercept | 29.6568 | 1 | < **0.0001** |
|  | Participant group | 11.4417 | 1 | **0.00072** |
|  | Participant’s age | 10.2095 | 1 | **0.00140** |
|  | Stimulus condition | 5.4567 | 1 | **0.01949** |
|  | Participant group x Participant’s age | 0.0028 | 1 | 0.95786 |
|  | Participant group x Stimulus condition | 8.5675 | 1 | **0.00342** |
|  | Participant’s age x Stimulus condition | 5.3752 | 1 | **0.02043** |
|  | Participant group x Participant’s age x Stimulus condition | 2.6091 | 1 | 0.10625 |

The tested models are similar to those presented in Additional File 7: Table S7 but include stimulus condition and all its interactions with participant’s age and group as additional fixed effects. Significance of the fixed effects is assessed using analysis of variance type III sum of squares and the Wald χ^2^ test. *p*‑values below 0.05 are highlighted in bold.

Abbreviations: df: degrees of freedom; ROI: region-of-interest.
