# Supplementary material for: Social attention to activities in children and adults with autism spectrum disorder: effects of context and age
Source: Mol Autism. 2020 Oct 19;11:79. doi: 10.1186/s13229-020-00388-5 (PMC7574440; doi:10.1186/s13229-020-00388-5)
Supplement: Supplementary file 17 — Table S13. Fixed effects in the linear mixed-effect model that compare slopes of the relationships between participant’s age and % looking time between the two groups of participants while accounting for the effects of stimulus condition and region of interest. The tested model is similar to those presented in Additional file 7: Table S7 and Additional file 16: Table S12 but includes stimulus condition and region of interest as well as all interactions between the latter two factors and participant’s age and group as additional fixed effects. Only data of the ROIs Activity and Heads are analyzed. Significance of the fixed effects is assessed using analysis of variance type III sum of squares and the Wald χ2 test. p values below 0.05 are highlighted in bold. df degrees of freedom, ROI region-of-interest. [file 13229_2020_388_MOESM17_ESM.docx]

**Table S13.** Fixed effects in the linear mixed-effect model that compare slopes of the relationships between participant’s age and % looking time between the two groups of participants while accounting for the effects of stimulus condition and region of interest.

| Fixed effect | χ^2^-statistic | df | *p*-value |
| --- | --- | --- | --- |
| Intercept | 841.8651 | 1 | < **0.0001** |
| Participant group | 1.8336 | 1 | 0.17571 |
| Participant’s age | 11.9752 | 1 | **0.00054** |
| Stimulus condition | 1.5153 | 1 | 0.21833 |
| ROI | 289.5194 | 1 | < **0.0001** |
| Participant group x Participant’s age | 0.3679 | 1 | 0.54417 |
| Participant group x Stimulus condition | 0.7831 | 1 | 0.37619 |
| Participant group x ROI | 7.6642 | 1 | **0.00563** |
| Participant’s age x Stimulus condition | 3.9652 | 1 | **0.04645** |
| Participant’s age x ROI | 16.1265 | 1 | < **0.0001** |
| Stimulus condition x ROI | 2.1871 | 1 | 0.13917 |
| Participant group x Participant’s age x Stimulus condition | 0.0068 | 1 | 0.93446 |
| Participant group x Participant’s age x ROI | 0.4666 | 1 | 0.49457 |
| Participant group x Stimulus condition x ROI | 2.1922 | 1 | 0.13871 |
| Participant’s age x Stimulus condition x ROI | 3.9846 | 1 | **0.04592** |
| Participant group x Participant’s age x Stimulus condition x ROI | 0.2089 | 1 | 0.64767 |

The tested model is similar to those presented in Additional File 7: Table S7 and Additional File 16: Table S12 but includes stimulus condition and region of interest as well as all interactions between the latter two factors and participant’s age and group as additional fixed effects. Only data of the ROIs *Activity* and *Heads* are analyzed. Significance of the fixed effects is assessed using analysis of variance type III sum of squares and the Wald χ^2^ test. *p*‑values below 0.05 are highlighted in bold. Abbreviations: df: degrees of freedom; ROI: region-of-interest.
