# Supplementary material for: Social attention to activities in children and adults with autism spectrum disorder: effects of context and age
Source: Mol Autism. 2020 Oct 19;11:79. doi: 10.1186/s13229-020-00388-5 (PMC7574440; doi:10.1186/s13229-020-00388-5)
Supplement: Supplementary file 18 — Table S14. Relationship between participant’s age and % looking time for the ROIs Activity and Heads tested for the groups of participants below a specified age. Only data of the participants below a specified age are analyzed, with n indicating their total number for each of the two stimulus conditions and groups of participants separately. rS corresponds to a Spearman partial correlation coefficient computed on the data of both groups of participants for each selection of participants and stimulus condition separately. The corresponding two-sided p value is shown in parentheses. CI corresponds to a 95% equal-tailed two-sided confidence interval for the computed correlation coefficient. p values below 0.05 and confidence intervals that do not include 0 are highlighted in bold. ASD autism spectrum disorder, CI confidence interval, ROI region-of-interest, TD typically developing [file 13229_2020_388_MOESM18_ESM.docx]

**Table S14.** Relationship between participant’s age and % looking time for the ROIs *Activity* and *Heads* tested for the groups of participants below a specified age.

| ROI | Age, years | Statistic | Shared focus | | Mutual gaze | |
| --- | --- | --- | --- | --- | --- | --- |
|  |  |  | ASD | TD | ASD | TD |
| Activity | ≤ 40 | n | 118 | 37 | 105 | 36 |
|  |  | r_S_ (*p*-value) | -0.127 (0.115) | | **-0.378 (< 10^-5^)** | |
|  |  | 95% CI | (-0.286; 0.020) | | **(-0.546; -0.243)** | |
|  | ≤ 35 | n | 117 | 35 | 104 | 34 |
|  |  | r_S_ (*p*-value) | -0.102 (0.213) | | **-0.345 (< 10^-4^)** | |
|  |  | 95% CI | (-0.264; 0.060) | | **(-0.508; -0.202)** | |
|  | ≤ 30 | n | 115 | 34 | 103 | 33 |
|  |  | r_S_ (*p*-value) | -0.098 (0.236) | | **-0.360 (< 10^-4^)** | |
|  |  | 95% CI | (-0.257; 0.057) | | **(-0.514; -0.228)** | |
|  | ≤ 25 | n | 110 | 34 | 99 | 33 |
|  |  | r_S_ (*p*-value) | -0.090 (0.286) | | **-0.377 (< 10^-5^)** | |
|  |  | 95% CI | (-0.247; 0.075) | | **(-0.530; -0.245)** | |
|  | ≤ 20 | n | 105 | 34 | 95 | 33 |
|  |  | r_S_ (*p*-value) | -0.074 (0.390) | | **-0.378 (< 10^-4^)** | |
|  |  | 95% CI | (-0.247; 0.086) | | **(-0.541; -0.242)** | |
| Heads | ≤ 40 | n | 118 | 37 | 105 | 36 |
|  |  | r_S_ (*p*-value) | 0.083 (0.304) | | **0.274 (0.01)** | |
|  |  | 95% CI | (-0.067; 0.242) | | **(0.124; 0.446)** | |
|  | ≤ 35 | n | 117 | 35 | 104 | 34 |
|  |  | r_S_ (*p*-value) | 0.044 (0.589) | | **0.239 (0.01)** | |
|  |  | 95% CI | (-0.113; 0.209) | | **(0.087; 0.407)** | |
|  | ≤ 30 | n | 115 | 34 | 103 | 33 |
|  |  | r_S_ (*p*-value) | 0.029 (0.730) | | **0.235 (0.01)** | |
|  |  | 95% CI | (-0.128; 0.195) | | **(0.068; 0.404)** | |

Continued on the following page.

| ROI | Age, years | Statistic | Shared focus | | Mutual gaze | |
| --- | --- | --- | --- | --- | --- | --- |
|  |  |  | ASD | TD | ASD | TD |
| Heads | ≤ 25 | n | 110 | 34 | 99 | 33 |
|  |  | r_S_ (*p*-value) | 0.034 (0.688) | | **0.280 (0.01)** | |
|  |  | 95% CI | (-0.121; 0.198) | | **(0.114; 0.449)** | |
|  | ≤ 20 | n | 105 | 34 |  |  |
|  |  | r_S_ (*p*-value) | 0.040 (0.638) | | **0.308 (< 10^-3^)** | |
|  |  | 95% CI | (-0.131; 0.199) | | **(0.157; 0.476)** | |

Only data of the participants below a specified age are analyzed, with n indicating their total number for each of the two stimulus conditions and groups of participants separately. r_S_ corresponds to a Spearman partial correlation coefficient computed on the data of both groups of participants for each selection of participants and stimulus condition separately. The corresponding two-sided *p*-value is shown in parentheses. CI corresponds to a 95% equal-tailed two-sided confidence interval for the computed correlation coefficient. *p*‑values below 0.05 and confidence intervals that do not include 0 are highlighted in bold.

Abbreviations: ASD: autism spectrum disorder; CI: confidence interval; ROI: region-of-interest; TD: typically developing.
