# Supplementary material for: Social attention to activities in children and adults with autism spectrum disorder: effects of context and age
Source: Mol Autism. 2020 Oct 19;11:79. doi: 10.1186/s13229-020-00388-5 (PMC7574440; doi:10.1186/s13229-020-00388-5)
Supplement: Supplementary file 19 — Table S15. Fixed effects in linear mixed-effects models comparing slopes of the relationships between participant’s age and % looking time across the two groups of participants that are below a specified age. Significance of the fixed effects is assessed using analysis of variance type III sum of squares and the Wald χ2 test. p values below 0.05 are highlighted in bold. df degrees of freedom, ROI region-of-interest. [file 13229_2020_388_MOESM19_ESM.docx]

**Table S15.** Fixed effects in linear mixed-effects models comparing slopes of the relationships between participant’s age and % looking time across the two groups of participants that are below a specified age.

| ROI | Fixed effect | χ^2^-statistic | df | *p*-value |
| --- | --- | --- | --- | --- |
| Participants ≤ 40 years | | | | |
| Activity | Intercept | 630.4297 | 1 | **< 10^-15^** |
|  | Participant group | 0.1906 | 1 | 0.66243 |
|  | Participant’s age | 2.8623 | 1 | 0.09068 |
|  | Participant group x Participant’s age | 2.82161 | 1 | 0.093 |
| Heads | Intercept | 34.9066 | 1 | **< 10^-8^** |
|  | Participant group | 0.8230 | 1 | 0.36429 |
|  | Participant’s age | 4.5897 | 1 | **0.03216** |
|  | Participant group x Participant’s age | 3.1409 | 1 | 0.07635 |
| Participants ≤ 35 years | | | | |
| Activity | Intercept | 567.6494 | 1 | **< 10^-15^** |
|  | Participant group | 1.5448 | 1 | 0.21390 |
|  | Participant’s age | 1.5301 | 1 | 0.26109 |
|  | Participant group x Participant’s age | 4.9388 | 1 | **0.02626** |
| Heads | Intercept | 30.4955 | 1 | **< 10^-7^** |
|  | Participant group | 1.2463 | 1 | 0.26426 |
|  | Participant’s age | 4.3707 | 1 | **0.03656** |
|  | Participant group x Participant’s age | 0.5443 | 1 | 0.46064 |
| Participants ≤ 30 years | | | | |
| Activity | Intercept | 534.3098 | 1 | **< 10^-15^** |
|  | Participant group | 1.3258 | 1 | 0.24956 |
|  | Participant’s age | 2.44131 | 1 | 0.11818 |
|  | Participant group x Participant’s age | 3.0791 | 1 | 0.07931 |

Continued on the following page.

| ROI | Fixed effect | χ^2^-statistic | df | *p*-value |
| --- | --- | --- | --- | --- |
| Heads | Intercept | 26.8478 | 1 | **< 10^-6^** |
|  | Participant group | 0.1192 | 1 | 0.72995 |
|  | Participant’s age | 3.8361 | 1 | 0.05016 |
|  | Participant group x Participant’s age | 1.0227 | 1 | 0.31188 |
| Participants ≤ 25 years | | | | |
| Activity | Intercept | 431.9118 | 1 | **< 10^-15^** |
|  | Participant group | 1.0720 | 1 | 0.3005 |
|  | Participant’s age | 2.5414 | 1 | 0.1109 |
|  | Participant group x Participant’s age | 2.6042 | 1 | 0.1066 |
| Heads | Intercept | 17.4256 | 1 | **< 10^-4^** |
|  | Participant group | 0.2980 | 1 | 0.58511 |
|  | Participant’s age | 5.2577 | 1 | **0.02185** |
|  | Participant group x Participant’s age | 0.6177 | 1 | 0.43191 |
| Participants ≤ 20 years | | | | |
| Activity | Intercept | 349.061 | 1 | **< 10^-15^** |
|  | Participant group | 0.9188 | 1 | 0.3378 |
|  | Participant’s age | 2.1742 | 1 | 0.1403 |
|  | Participant group x Participant’s age | 2.2856 | 1 | 0.1306 |
| Heads | Intercept | 9.8266 | 1 | **0.001720** |
|  | Participant group | 0.6587 | 1 | 0.417016 |
|  | Participant’s age | 7.4569 | 1 | **0.006319** |
|  | Participant group x Participant’s age | 0.2193 | 1 | 0.639605 |

Data for each ROI are pooled across the two stimulus conditions. Significance of the fixed effects is assessed using analysis of variance type III sum of squares and the Wald χ^2^ test. *p*‑values below 0.05 are highlighted in bold.

Abbreviations: df: degrees of freedom; ROI: region-of-interest.
