# Supplementary material for: Social attention to activities in children and adults with autism spectrum disorder: effects of context and age
Source: Mol Autism. 2020 Oct 19;11:79. doi: 10.1186/s13229-020-00388-5 (PMC7574440; doi:10.1186/s13229-020-00388-5)
Supplement: Supplementary file 20 — Table S16. Mean % looking time for each individual region of interest, stimulus condition, group of participants and clinical site. n indicates the number of analyzed participants. ASD autism spectrum disorder, TD typically developing. [file 13229_2020_388_MOESM20_ESM.docx]

**Table S16.** Mean % looking time for each individual region of interest, stimulus condition, group of participants and clinical site.

| Region of interest | | Activity | | | | Heads | | | | Bodies | | | | Background | | | |
| --- | --- | --- | --- | --- | --- | --- | --- | --- | --- | --- | --- | --- | --- | --- | --- | --- | --- |
| Site no. | Condition | Shared focus | | Mutual gaze | | Shared focus | | Mutual gaze | | Shared focus | | Mutual gaze | | Shared focus | | Mutual gaze | |
|  | Group | ASD | TD | ASD | TD | ASD | TD | ASD | TD | ASD | TD | ASD | TD | ASD | TD | ASD | TD |
| 1 | n | 1 | 0 | 0 | 0 | 1 | 0 | 0 | 0 | 1 | 0 | 0 | 0 | 1 | 0 | 0 | 0 |
|  | mean | 58.0 | - | - | - | 19.5 | - | - | - | 4.6 | - | - | - | 18.0 | - | - | - |
| 2 | n | 14 | 3 | 14 | 3 | 14 | 3 | 14 | 3 | 14 | 3 | 14 | 3 | 14 | 3 | 14 | 3 |
|  | mean | 55.6 | 58.2 | 55.5 | 57.8 | 15.2 | 14.3 | 12.4 | 15.9 | 9.0 | 7.7 | 9.9 | 7.2 | 20.3 | 19.8 | 22.1 | 19.2 |
| 3 | n | 5 | 2 | 5 | 2 | 5 | 2 | 5 | 2 | 5 | 2 | 5 | 2 | 5 | 2 | 5 | 2 |
|  | mean | 66.5 | 66.2 | 60.0 | 67.5 | 14.3 | 20.3 | 13.6 | 12.7 | 3.0 | 3.0 | 7.3 | 5.0 | 16.2 | 10.5 | 19.2 | 14.8 |
| 4 | n | 18 | 7 | 19 | 7 | 18 | 7 | 19 | 7 | 18 | 7 | 19 | 7 | 18 | 7 | 19 | 7 |
|  | mean | 59.8 | 61.9 | 54.3 | 53.4 | 16.9 | 22.0 | 18.2 | 26.2 | 5.0 | 5.0 | 7.1 | 6.6 | 18.3 | 11.1 | 20.4 | 13.9 |
| 5 | n | 17 | 6 | 13 | 6 | 17 | 6 | 13 | 6 | 17 | 6 | 13 | 6 | 17 | 6 | 13 | 6 |
|  | mean | 59.2 | 51.4 | 57.6 | 46.9 | 14.2 | 25.7 | 13.8 | 30.8 | 8.0 | 6.4 | 8.2 | 7.0 | 18.7 | 16.5 | 20.4 | 15.4 |
| 6 | n | 15 | 4 | 12 | 4 | 15 | 4 | 12 | 4 | 15 | 4 | 12 | 4 | 15 | 4 | 12 | 4 |
|  | mean | 57.1 | 58.3 | 53.4 | 49.0 | 18.3 | 19.6 | 18.5 | 23.0 | 7.8 | 5.3 | 8.7 | 9.0 | 16.7 | 16.8 | 19.5 | 19.0 |
| 7 | n | 8 | 3 | 7 | 3 | 8 | 3 | 7 | 3 | 8 | 3 | 7 | 3 | 8 | 3 | 7 | 3 |
|  | mean | 51.4 | 63.5 | 58.4 | 62.2 | 18.2 | 19.9 | 13.8 | 20.9 | 6.7 | 5.0 | 11.0 | 5.5 | 23.7 | 11.6 | 16.8 | 11.5 |
| 8 | n | 19 | 6 | 15 | 5 | 19 | 6 | 15 | 5 | 19 | 6 | 15 | 5 | 19 | 6 | 15 | 5 |
|  | mean | 59.9 | 53.2 | 59.7 | 42.1 | 15.4 | 24.5 | 16.7 | 32.8 | 6.9 | 5.8 | 7.5 | 8.0 | 17.8 | 16.5 | 16.1 | 17.1 |
| 9 | n | 23 | 9 | 22 | 9 | 23 | 9 | 22 | 9 | 23 | 9 | 22 | 9 | 23 | 9 | 22 | 9 |
|  | mean | 60.7 | 49.5 | 59.9 | 42.0 | 13.5 | 24.8 | 13.1 | 28.3 | 8.3 | 8.9 | 9.3 | 7.7 | 17.5 | 16.9 | 17.7 | 22.1 |

n indicates the number of analyzed participants. Abbreviations: ASD: autism spectrum disorder; TD: typically developing.
